# Repositioning Cannabinoids and Terpenes as Novel EGFR-TKIs Candidates for Targeted Therapy Against Cancer: A virtual screening model using CADD and biophysical simulations

**DOI:** 10.1016/j.heliyon.2023.e15545

**Published:** 2023-04-17

**Authors:** Ossama Daoui, Suraj N. Mali, Kaouakeb Elkhattabi, Souad Elkhattabi, Samir Chtita

**Affiliations:** a Laboratory of Engineering, Systems and Applications, National School of Applied Sciences, Sidi Mohamed Ben Abdellah-Fez University, P.O. Box 72, Fez, Morocco; b Department of Pharmaceutical Sciences and Technology, Birla Institute of Technology, Mesra, India, 835215; c Department of Fundamental Sciences, Faculty of Dental Medicine, Mohammed V University in Rabat, Morocco; d Laboratory of Analytical and Molecular Chemistry, Faculty of Sciences Ben M'Sik, Hassan II University of Casablanca, P.O. Box 7955, Casablanca, Morocco

**Keywords:** *Cannabis sativa* L., Virtual Screening, CADD, Semi-flexible & Flexible Molecular Docking, MM-GBSA free Binding energy, The inhibition constant (Ki), Molecular Dynamics, EGFR-TKIs, Breast & Lung Cancer, Cannabinoids & Terpenes

## Abstract

This study examines the potential of *Cannabis sativa* L. plants to be repurposed as therapeutic agents for cancer treatment through designing of hybrid Epidermal growth factor receptor tyrosine kinase inhibitors (EGFR-TKIs). A set of 50 phytochemicals was taken from Cannabinoids and Terpenes and subjected for screening using Semi-flexible and Flexible Molecular Docking methods, MM-GBSA free binding energy computations, and pharmacokinetic/pharmacodynamic (ADME-Tox) predictions. Nine promising phytochemicals, Cannabidiolic acid (CBDA), Cannabidiol (CBD), Tetrahydrocannabivarin (THCV), Dronabinol (Δ-9-THC), Delta-8-Tetrahydrocannabinol (Δ-8-THC), Cannabicyclol (CBL), Delta9-tetrahydrocannabinolic acid (THCA), Beta-Caryophyllene (BCP), and Gamma-Elemene (γ-Ele) were identified as potential EGFR-TKIs natural product candidates for cancer therapy. To further validate these findings, a set of Molecular Dynamics simulations were conducted over a 200 ns trajectory. This hybrid early drug discovery screening strategy has the potential to yield a new generation of EGFR-TKIs based on natural cannabis products, suitable for cancer therapy. In addition, the application of this computational strategy in the virtual screening of both natural and synthetic chemical libraries could support the discovery of a wide range of lead drug agents to address numerous diseases.

## Highlights

• Cannabinoids & Terpenes derived from Cannabis sativa L. screened in silico for targeting cancer via inhibiting the EGFR-TKD enzyme.
• Hybrid approach of structure-based & biophysical simulations to identify EGFR-TKIs..
• Semi-flexible & Flexible Molecular Docking methods, Drug-like, ADME-Tox, MM-GBSA and MD used to identify stable EGFR-TKIs.
• The amino acid residues of Leucine, Phenylalanine, Valine, Lysine, Cysteine, & Threonine targeted for cancer cell line inhibition.
• In silico drug discovery identified new generation of EGFR-TKIs as candidates for target therapy.
• Natural substances from Cannabis sativa L. repurposed for therapeutics.

## Graphical abstract

**Figure:**
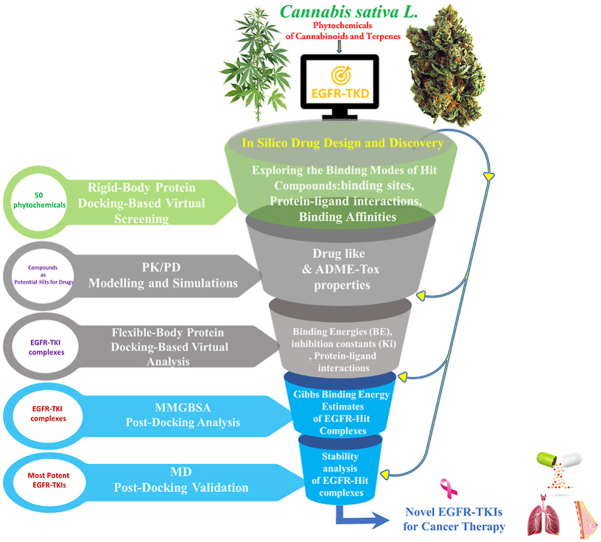

## 1 Introduction

Despite the fact that the *Cannabis sativa* L. plant has many uses, this plant is not without its utilitarian benefits, especially in fields of medicinal and pharmaceutical applications. It has therefore become a necessity to explore benefits of this plant by legalizing its use for the medical and industrial purposes [1]. The *Cannabis sativa* L. plant was originally popular in East Asia, but it has now become a popular plant all over the world due to the development of cultivation types [2]. Throughout a history, it has been cultivated and used as a source of artificial fiber, amusement, food, mood enhancement, and also in traditional medicine [3,4]. Two countries, Morocco and Afghanistan, are among the largest producers of hashish obtained from the cannabis plant, which is being distributed and consumed worldwide for its psychological effects [[5], [6], [7]]. Cannabis has already been found to have certain biological activities that could allow it to be used for medicinal purposes. Among the permitted therapeutic potentials, it mentions the ability to regulate sleep disorders [8], inflammation [9], neuro-degeneration [10], pain [11], nausea [12], anorexia [13], diseases of cancer and epilepsy [14].

Although the main psychoactive substance in cannabis is a Tetrahydrocannabinol (THC) [15], the cannabis plant contains several other active cannabinoids, including cannabis isolated (>120) such as Cannabidiol (CBD), Cannabinol (CNB), Terpenes (>120) [16,17], Flavonoids (∼34), and Phenolic compounds (∼42), etc. [1,[18], [19], [20]]. There are several categories of cannabis, the most important of which is the one based on the rate of THC and CBD and CNB [21]. The higher the THC level, the more the product belongs to the dope class, while the higher the CBD and CNB level, the more the product belongs to the cannabinoid class [22,23]. It should be noted that both Cannabidiol (CBD) and Cannabinol (CBN) do not have a psychoactive effect, but can block the effect of THC on the nervous system [23]. Although the THC (PubChem CID: 16078), and CBD (PubChem CID: 644019) and CNB (PubChem CID:2543) come from the same plant source, they have different biological effects on humans. The reason for these different therapeutic effects may be due to the structural diversities of these phytochemicals derived from cannabis as well as the specific cultivar of which they are derived [24]. This diversity is one of the rationales for studying differences between natural cannabis products and evaluating their effects on pharmacological properties *in silico*, *in vitro* and *in vivo*. In the context of the legalization of the use of cannabis products in the medical field, the biological activity of phytochemicals isolated from cannabis has been examined under different conditions, and here we highlight some effects of seven isolated cannabis include (Δ^9^-Tetrahydrocannabinol, Cannabidiol, Cannabigerol, Cannabichromene, Cannabidivarin, Tetrahydrocannabivirin) against Breast Cancer, Brain Cancer, Leukemia, Lung Cancer, Melanoma & Myeloma, Hepatocellular Carcinoma, Pancreatic, Prostate, Colon Cancer, Endometrial, Cervival, Oral Cancer, Glioma & Neuroblastoma, Cervical, Endometrial and Ovarian Cancer, etc. [25]. Among the seven cannabinoids reviewed, only Δ^9^-Tetrahydrocannabinol and Cannabidiol were able to reach clinical trials against brain cancer, leukemia, glioma cancer and neuroblastoma cancer [25]. While the rest of the isolated cannabis did not exceed the phase of *in vivo* testing as the highest estimate against the other types of cancer listed in the current review [25]. The reason for this could be due to the occurrence of undesirable side effects in living organisms submitted to the trial. Although the various functions of several bioactive compounds in cannabis have been reported to be beneficial against cancer, there is an almost complete lack of evaluation of the bioactivity of other natural products found in cannabis, such as the cannabinoids and terpenes. Due to the availability of wide varieties of natural products such as capsules, oils, extracts and tinctures based on terpenes, other medicinal applications of these agents are still unknown or have not yet been reported, especially their potential therapeutic against cancer. From this background, it will be the basis of our investigation to predict *in silico* the prospects of using terpenes and cannabinoids isolated for breast and lung cancer as a targeted model.

Due to the lack of efficient treatments accessible to all populations at the lowest cost and with less toxicity, efforts of researchers have turned towards medicinal plants such as *Cannabis sativa* L. and their therapeutic benefits against breast and lung cancer [[26], [27], [28]]. The World Health Organization (WHO) reported 10.44 million cancer deaths in 2020, including 2.26 million cases of breast cancer, 2.21 million cases of lung cancer, 1.93 million cases of colon and rectal cancer, 1.41 million cases of prostate cancer, 1.20 million cases of non-melanoma skin cancer, and 1.09 million cases of stomach cancer [[29], [30], [31]].

Despite tremendous progress in the cancer treatment methods around the world, the prospect of turning cancer into a disease that can be lived with or completely treated remains a long way off. However, this reality is regarded as a strong motivator for researchers in their various disciplines related to this context, with the horizon of achieving the desired goal. This is demonstrated by the wide range of available cancer therapies, including hormonal therapy, radiotherapy, surgery, targeted therapy with drugs/vaccines, gene therapy, and other chemotherapy treatments [32]. In this regard, targeted molecular therapies based on interference/interaction with specific molecular receptors such as proteins/enzymes have revolutionized the treatment of a variety of cancer diseases, including lung, breast, colorectal, and ovarian cancer, as a means of preventing cancer growth [33].

In this progression, The epidermal growth factor receptor (EGFR) tyrosine kinase domain (TKD) targeted therapies point outs a new era in precision oncology [34,35]. This is because the EGFR (or EGFR; ErbB-1; HER1) plays critical roles in the maintenance of epithelial tissue as well as the control of cell division and survival [36]. However, the receptor regulates many signaling pathways in the body, with the absence or low expression of EGFR and other receptor tyrosine kinases in humans leading to many diseases such as Alzheimer's disease [37,38], while overexpression of EGFR-TKD is linked to the development of many tumor diseases, including breast and lung cancers, which are currently the most lethal [35].

Focusing on ways to treat cancer tumors resulting from overexpression of the EGFR receptor tyrosine kinase, tyrosine kinase inhibitors (TKIs)have been and remain a successful therapeutic key in this regard [39,40]. Several EGFR therapies have been approved by the FDA for NSCLC and breast cancer other cancers, including Erlotinib and Gefitinib (first-generation TKIs); Afatinib, Dacomitinib, and Neratinib (second-generation TKIs); Osimertinib, Afatinib, and Lazertinib (third-generation TKIs); JBJ-0412502, BI, BI-4020 (fourth-generation TKIs) [35,41,42]. Combining chemotherapy with the targeted therapy through EGFR-targeted by the small molecule TKIs is emerging as a very important therapeutic model in the cancer treatment scenarios to achieve a longer life expectancy for patients. However, TKIs face many challenges, including a relatively limited range of targeted cancers, drug resistance after long-term treatment, and potential toxicity risks [43]. These reasons are sufficient to multiply studies and researches to develop and to screen new generations of EGFR-TKIs for an effective cancer treatment. In this regard, it appears that most FDA-approved EGFR-TKIs are based on small molecules, with the exception of some inhibitors such as Erlotinib (Tarceva) and Gefitinib (Iressa) which are based on phytochemicals *Taxus brevifolia and Ancaria tomentosa,* respectively [44,45]. In this context, four phytochemicals Curcumin [46], derived from *Curcuma longa*; Resveratrol [47], from Vitis *vinifera*; Quercetin [48], from *Sophora japonica*; and Pepperlongumene [49], from *Piper longum*, were studied and tested against cancer as a part of the EGFR-TKIs class. These compounds have demonstrated potential in their ability to inhibit/regulate EGFR-TKD activity and reduce resistance to several of its standard drug inhibitors. This highlights the need to expand the existing databases of prospective phytochemical agents for their use as EGFR-TKIs, through further research into different and diverse categories of natural products. With this in mind, the present study has focused on an untapped potential of *Cannabis sativa* L. based phytochemicals to develop a new generation of EGFR-TKIs with therapeutic applications for cancer and other diseases that are currently untreatable. With this insight, recent research has revealed that cannabis extracts may have potential for treating cancer [50]. Cannabinoids such as Cannabidiol (CBD), Cannabigerol (CBG), and Cannabinol (CNB) have been tested for their ability to bind to and inhibit the Epidermal Growth Factor Receptor (EGFR), which is associated with cancer progression [50]. This may be a turning point in the discovery of novel *Cannabis sativa L*-based drugs that specifically target cancers brought on by the overexpression of EGFR. Therefore, shifting research interest towards *Cannabis sativa L*-derived phytochemicals in the development of cancer therapies is a paradigm that merits further attention. With our study, we hope to promote this paradigm.

In light of these scenarios, consider the frightening statistics for breast and lung cancer deaths worldwide, which have been reported to account for nearly 50% of all deaths [51]. Because of the high deaths caused by these two cancers, our current research focuses on them as models for exploring potential therapeutic ways to reduce the rate of death caused by these cancers using phytochemicals isolated from *Cannabis sativa* L plant. To achieve this goal, we collected a database of 50 samples of cannabis extracts that were statistically categorized as the optimal extracts among the 279 samples statistically analyzed in a study conducted by Matan Birenboim et al. [1]. In the current study, we categorized the 50 phytochemical compounds into two sets. The first set consisted of 12 cannabinoids (**C1**–**C12**) and the second set consisted of 38 terpenes (**T1**-**T38**) (Table S1).

Based on molecular modeling techniques and their importance in computer-aided drug design (CADD) [52,53], we performed a comprehensive screening of 50 phytochemicals to determine their bioavailability, pharmacokinetics/pharmacodynamics, and their likely interactions with the Epidermal growth factor receptor tyrosine kinase domain (EGFR-TKD) involved in the proliferation of breast and lung cancer cell lines and several other types of cancer [[54], [55], [56]]. Based on predictions of drug-likeness and toxicity risks features, *in silico* ADME-Tox (Absorption, Distribution, Metabolism, Excretion and Toxicity) modeling, Molecular Docking Semi-Flexible& Flexible, theoretical values of inhibitory constants (Ki), MM-GBSA free binding energies, and Molecular Dynamics analysis, we screened top lead molecular scaffolds that would be best likely to inhibit breast and lung cancer cell growth by targeting the EGFR-TKD. To facilitate the identification of new candidate phytochemicals agents for their use as inhibitors of breast and lung cancer by targeting the Epidermal Growth Factor Receptor Tyrosine Kinase Domain (EGFR-TKD). In our present study, we use two drugs Tamoxifen (PubChem CID: 2733526 and Erlotinib (PubChem CID: 176870) as references to construct the hypothetical screening and make the rational comparisons. This is due to the broad clinical application of Erlotinib and Tamoxifen in patients with lung and breast cancer [57].

## 2 Material and methods

### 2.1 Identification of targeted receptor and standard drugs for drug design

The epithelial growth factor receptor (EGFR) mediates several tyrosine signaling pathways to transmit signals to the exterior/interior of the cell to perturb the cell function. The EGFR (Epidermal Growth Factor Receptor) is a member of the ErbB family of receptor tyrosine kinases [58]. The ErbB family consists of four receptor tyrosine kinases, including EGFR (ErbB1), ErbB2 (HER2), ErbB3 and ErbB4 [59]. The EGFR is the major receptor of the ErbB family and is responsible for transmitting signals from the cell surface ligands to an inside of the cell. It is involved in a variety of physiological processes, including cell growth, differentiation, migration, and survival [60]. In this regard, first and second generations of tyrosine kinase inhibitors (TKIs), the most important of which is Erlotinib, have often been a good and broad treatment alternative for several types of cancer including lung cancer through targeting EGFR-TKD. However, cases of resistance to these inhibitors leads to hyper-phosphorylation, enzyme overexpression, and the generation of new EGFR-TKD mutations [61]. Therefore, the patient resistance to this inhibitor limits its therapeutic efficacy, adversely affecting desired therapeutic endpoints. This emphasizes the need to discover stronger and more efficient alternatives to the conventional EGFR-TKIs, like erlotinib, which is used as a standard drug therapy for lung cancer in this study.

As for the treatment of breast cancer with EGFR-TKIs, some drugs such as lapatinib, Neratinib and Pyrotinib have already been shown significant clinical efficacy, but they are not unilateral and must be combined with other therapeutic agents [62]. Thus, the potential of EGFR-TKIs in the breast cancer treatment needs a further study and refinement until more suitable agents are found for the dual use, alone or in the combination with other therapies. Tamoxifen has been used as an effective anti-estrogen drug treatment for breast cancer for the last four decades. Tamoxifen inhibits estrogen (ER), which results in a significant downregulation of the ERBB2 protein, limiting the effects of estrogen in most areas of the body, including the breast [63,64]. Tamoxifen is still one of the most effective breast cancer treatments, reducing mortality by at least 30% [65]. However, resistance to this drug remains one of the major issues in the fight against breast cancer, as it has been observed that after the initial 5–10 year treatment period, breast cancer patients develop resistance to this drug [65,66]. In this context, a study conducted by Wang YK et al. [67] confirmed that ERBB2 and EGFR are membrane-bound tyrosine kinases with 95% similar structures, all of which, when overexpressed, can lead to cell transformation, cell proliferation and cancer. The high expression of ERBB2 has been linked to increased tumor invasiveness, metastasis, resistance to chemotherapy and poor prognosis. Another study, Tomoya T. et al. [68], reported that EGFR inhibition reduces tamoxifen resistance. As a result, the development of new potent EGFR inhibitors could provide a new therapeutic approach for breast cancer patients who have developed Tamoxifen resistance. These strong relationships between EGFR and ERBB2 in terms of Tamoxifen efficacy could make Tamoxifen an indirect pathway inhibitor of EGFR-TKD. More in-depth studies are needed to qualify this proposal. In this light, the repositioning of Tamoxifen as an ERBB2 inhibitor into a candidate EGFR-TKI could lead to further research and studies regarding the ability of this drug to inhibit the development of cancer cell lines resulting from EGFR overexpression, including breast cancer. This provision requires further and more extensive selective investigations. This is one of the main motivations for including this drug as a potential EGFR-TKI alongside Erlotinib in the current study. This insight may be useful in driving further research related to drug repositioning approaches.

### 2.2 Unlocking the potential of virtual screening: An exploration of methodology

• Selected Phytochemicals

This study used 50 phytochemicals derived from cannabinoids and Cannabis terpenes as inputs for early drug discovery using computational approaches to identify potential EGFR-TKIs candidate drugs for cancer therapy. For this, we adopted the Structure-Based Drug Design strategy as an input to implement this *in silico* computational study. In the light of this, our basic stage is to use a hybrid strategy based on the Molecular Docking approach to investigate the affinity and structural compatibility of the EGFR protein with the fifty cannabinoid/terpene phytochemicals. Table S1 in the supplementary information lists the identification details of these substances available in the PubChem library, including their chemical names, 2D structures, and ID codes.

• Docking-Based Virtual Screening Strategy

Bioinformatics techniques have been successfully implemented in the drug design and discovery pipeline, resulting in many successes. These techniques allow for quick screening and prediction of compounds' biological activity, making them especially attractive to the pharmaceutical industry. Molecular docking is a particularly important application of these techniques in the virtual screening [69,70]. Automated docking tools such as HADDOCK, Z-Dock, Molecular Operating Environment (MOE), PyRx, and AutoDock have been developed for various applications. AutoDock is specifically designed to dock small molecules to a protein receptor, while Z-Dock and HADDOCK are tailored for predicting protein-protein docking poses [71]. In this study, we implemented the virtual screening platform by utilizing rigid and flexible Molecular Docking with Autodock Vina 1.1.2 and Autodock 4. These two tools rely upon genetic algorithms (GA) and empirical scoring function (SF) to accurately predict potential drug candidates from large sets of compounds [72,73]. By harnessing the computational power of modern computers, Autodock Vina and Autodock 4 can quickly identify novel molecules with high binding affinity which may have previously gone unnoticed. The combination of genetic algorithms and scoring functions make virtual screening (VS) using Autodock Vina and Autodock 4 powerful tools for drug discovery and development. On this basis, we applied this hybrid Molecular docking approach to construct a VS model, in order to increase the affinity between the ligand and target protein, as well as to identify the ideal orientation and conformation of the ligand in the target protein's active binding pocket. We leveraged Semi-flexible and Flexible hybrid molecular docking methodology to construct a virtual screening model that would improve the binding between the ligand and the target protein, and identify the orientation and optimal conformation of the ligand in the active binding pocket of the target protein (for more information, see the supplementary information document).

• Assessing Crystal Structure Selection Using Criteria-Based Analysis

When selecting a crystal 3D structure from the Research Collaboratory for Structural Bioinformatics Protein Data Bank (RCSB PDB) for a CADD study, resolution, ligand flexibility, size, symmetry, composition, existing data or literature, available tools, extraction method and organism type should be taken into consideration [74,75]. High-resolution structures can provide exact details, while highly flexible ligands may not be suitable for Structure-Based Drug Design (SBDD) modeling. Large complexes tend to be more challenging to model, while complexes with fewer components may be simpler. Symmetry can simplify the analysis, and existing data or literature can help in performing *in silico* drug design modeling. Necessary tools should be available for *in silico* modeling, and the extraction method and type of organism should also be taken into account.

•Structure of the targeted protein

**Fig. 1:**
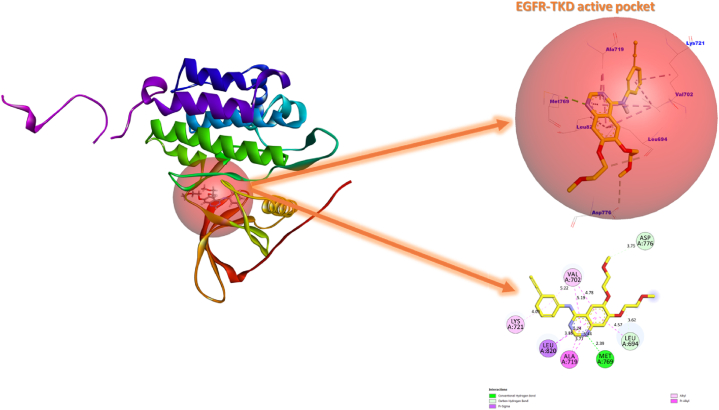
3D visualization of Erlotinib inhibitor in the active EGFR-TKD pocket.

The 3D crystal structure of EGFR complexed with Erlotinib (PDB code: 1M17) was obtained from the RCSB Protein Data Bank for this study (Fig. 1). The structure of EGFR in complex 1M17 consists of a single chain of 333 amino acids, with a molecular weight of 37.88 kDa, making it a suitable target for CADD-based studies. The small weight of the Erlotinib co-ligand (393.4 Da) is also beneficial for identifying an active site of interaction and the active amino acid residues in the EGFR-Erlotinib complex (1M17). Moreover, the experimental data such as Extraction Method (X-ray diffraction), Organism (*Homo sapiens*), Resolution (2.6 Å), R-Value Free (0.295), R-Value Work (0.251) and R-Value Observed (0.251), Expression System (*Escherichia coli*) further supports its suitability as an input for CADD-based studies [76]. In this study, we adopted the 3D crystal structure of the EGFR-Erlotinib complex (1M17) to implement a hypothetical assay based on molecular docking, MM-GBSA, and molecular dynamics simulations. The aim of this study was to assess the potential of phytochemical molecules to inhibit EGFR activity by using Erlotinib as a reference drug. This was in line with the subject of our current and previous studies [40,77], and it was also supported by the extensive use of this complex in the literature [76].

• Identifying Potential Tyrosine Kinase Inhibitors

The Erlotinib drug interactions in the EGFR active pocket of the 1M17 complex served as benchmarks in this study to identify candidate agents as novel EGFR-TKIs using molecular docking-based virtual screening [78,79]. We had selected Erlotinib as the standard drug in this study to identify and classify candidate molecules as EGFR-TKIs for lung cancer targeted therapy. However, given the critical importance of virtual screening based on molecular docking to reposition and target drugs for various therapeutic purposes, we included Tamoxifen as a standard drug to rank candidate molecules as EGFR-TKIs for targeted therapy against breast cancer. This ranking is established by comparing the binding energies of the examined phytochemical compounds with those of the standard drugs Tamoxifen and erlotinib in Protein-ligand complexes. Accordingly, the chance of a ligand binding to a protein increases with the amount of binding free energy produced by that protein's binding, lowering the complex's overall energy and making it more stable (more negative binding free energy). In light of this, we selected the ligands as prospective TKIs for dual-targeted therapy against lung and breast cancer that have more negative free binding energies than Erlotinib and Tamoxifen. Whereas, potential TKIs for targeted therapy against lung cancer includes ligands with more negative free binding energies compared to Erlotinib alone and less negative compared to Tamoxifen.

### 2.3 Preparation of the protein and the ligand

Using Molecular Operating Environment (MOE), Discovery Studio 2016, and AutoDockTools-1.5.6 tools, the EGFR-TKD protein structure was optimized and adapted for molecular docking simulation. During this step, missing side chains were added to the receptor backbone, polar hydrogens and a Gasteiger partial charge were added, water molecules and non-protein elements associated with the protein structure were also, removed. Using AUTOGRID based algorithms [80], the grid box coordinates of the ligand docking grid were determined in an accordance with the coordinates of the region occupied by the original Erlotinib ligand (4-anilinoquinazoline) inside the EGFR-TKD active pocket.

The depth of the XYZ input grid directions was fixed at 45 * 25 * 20 Å^3^ and the number of points in each direction were defined as x = 22.030 Å, y = 0.467 Å, z = 52.836 Å with a spacing of 0.375 Å. In this work, the seven rigid-flexible amino acid residues, Lysine (**Lys721)**, Valine (**Val702**), Methionine (**Met769**), Alanine (**Ala719)**, Leucine (**Leu694** and **Leu820)** and Aspartic (**Asp776**) with which Erlotinib interacts were used as references in the analysis of ligand-protein interaction patterns contributing to the inhibition of EGFR-TKD enzymatic activity (Fig. 1). Cannabinoids (C1–C12) and Terpenes (T1-T38) ligands, as well as Tamoxifen and Erlotinib ligand structures, were obtained from the National Library of Medicine (https://pubchem.ncbi.nlm.nih.gov).

All collected 50 ligands were re-modeled and optimized for their use as inputs in molecular docking simulations using SYBYL-X 2.1.1, GaussView 6.0, Gaussian 09W, AutoDockTools-1.5.6, and Discovery Studio 2016 [81].

### 2.4 Receptor design validation and mapping of active site coordinates

Given the importance of molecular modeling based on molecular docking in a computer-aided drug design approach, this protocol of docking must be initially validated. Therefore, we validated the structure of the EGFR-TKD (PDB ID:1M17) protein and the performance of the adopted molecular docking algorithms before proceeding to the second stage of simulations. To do this, we re-docked the Erlotinib ligand in the EGFR-TKD pocket as defined in Fig. 1, then matched the original ligand with the re-docked and evaluated the level of superimposition between them by computing the DockRMSD (root-mean-square deviation) value (https://zhanggroup.org/DockRMSD/).

When the RMSD value is < 2 Å, the adopted molecular docking approach is suggested to be valid and reliable in predicting protein-ligand interactions [82,83].

### 2.5 Developing a virtual screening model

Following the validation of the molecular docking protocol, we docked 50 cannabinoid and terpene ligands into the active pocket of EGFR-TKD. This was done to evaluate the affinity energies of the ligands towards the active amino acid residues in the EFGR-TKD pocket. In the present screening study, we examined protein-ligand interactions through a molecular docking procedure using the AutoDockVina 1.1.2 and AutoDock 4.2.6 packages, respectively [72,84]. This selection was adopted due to the accuracy and quality of the simulation of protein-ligand interactions provided by algorithms of these tools compared to other programs, particularly with respect to the affinity energies of ligands towards protein receptors and the prediction of the non-covalent interaction patterns [85].

#### 2.5.1 Autodock VINA virtual screening

During this screening, it should be noted that it is possible to perform a parallel set of semi-flexible molecular docking operations for multiple ligands to the same receptor and obtain separate data for each operation. Then, we inspected the output of the molecular docking by comparing the affinity energies of the ligand conformations to the active side chain residues of EGFR-TKD. In this systematic screening, we selected conformations (Flexible body ligands) whose affinity for the EGFR-TKD receptor (Rigid body protein) was more negative than or closer to that of the standard drugs, Tamoxifen and Erlotinib. In parallel, we eliminated EGFR-TKD incompatible phytochemicals that require very high binding energies to bind to them. This was done to ensure that we do not select structures that produce high affinities between the intermolecular and the receptor-ligand, which would likely cause an irreversible inhibition through the formation of covalent bonds (suicide inhibition). Whereas, we focused on ligands that produce low affinity (more negative binding energy) and lower force between intermolecular and receptor-ligand through the formation of non-covalent interactions (reversible inhibition).

#### 2.5.2 Analysis of lead compounds for drug-like properties and ADME-Tox effects

After completing the screening of phytochemicals for their affinity energies with EGFR-TKD, we proceeded further to study their drug-like profiles, their pharmacokinetic/pharmacodynamic (ADME-Tox) profiles. This is a routine procedure *in silico* screening because of its great importance in the drug design and discovery process before moving to the experimental and drug trials phases. In addition, this screening procedure is very important in predicting the toxicity risks of molecules and their adverse effects on the efficacy of candidate drug molecules. For example, the properties of Erlotinib, which has shown in *vitro*, *vivo* and *silico* a set of undesirable deviations in safe drugs, as a result, clinical trials of this drug have shown unsatisfactory results [86]. It is therefore necessary to evaluate the pharmacokinetic properties of drug molecules *in silico* before making their use available.

#### 2.5.3 Key criteria for lead compound selection

In this step, we identified candidate drug phytochemicals based on the detailed drug-like profiles of the desired bioavailability criteria of the drug molecules, based on the computational predictions provided via the SwissADME (http://www.swissadme.ch/index.php). Then, based on the pkCSM (https://www.ncbi.nlm.nih.gov/pmc/articles/PMC4434528/), we screened the ADME-Tox properties of the drug molecules that satisfied the bioavailability criteria proposed by Lipinski Rule of Five (RO5) and Viber rules [87]. In this regard, the most important parameters for which phytochemicals were evaluated for their lipophilicity (Log P), physicochemical properties (molecular weight, number of rotational bonds, number of H-bond acceptors and donors, Molar Refractivity), and medicinal chemistry (Bioavailability Score, synthetic accessibility) [87].

On the other hand, pharmacokinetic ADME-Tox parameters of phytochemicals were evaluated by predicting their absorption properties (skin permeability and human intestinal absorption) and distribution properties (human volume of distribution at steady state (VDss), unbound fraction, blood-brain barrier permeability (BBB), Central Nervous System permeability (CNS), metabolism properties (actions towards cytochrome P450 enzymes), excretion properties (Total Clearance index), toxicity parameters (AMES toxicity, Oral Rat Acute Toxicity (LD_50_), Hepatotoxicity, Maximum recommended tolerated dose). Drug-like and ADME-Tox predictions were supported by Osiris computations to evaluate the potential toxicity risks of selected phytochemicals (https://www.organic-chemistry.org/prog/peo/) [88].

Osiris computations provided molecular toxicity risk profiles, such as risks associated with mutagenicity, carcinogenicity, reproduction, and irritation that may be caused by undesirable radicals in the structure of SMILES-encoded molecules [89]. Once we had collected drug and pharmacodynamics property profiles of candidate molecules to inhibit EGFR-TKD enzymatic activity, we tested the binding interactions of these ligands to the active pocket of EGFR-TKD to confirm their safety. To achieve this goal, we followed the rational examination below.

### 2.6 Analysis of protein-ligand complex conformational stability

Despite the identification of drug-like and pharmacokinetics/pharmacodynamics properties of lead compounds, the pattern and requirements of the interaction of these compounds with target enzyme receptors remain a question that requires advanced biophysical modeling to solve. Therefore, in the present study, thus we decided to evaluate a set of helpful biophysical parameters to simplify the insight in this regard. For this purpose, we combined the following modelling techniques: Flexible Molecular Docking, Mechanized Generalized Surface Area (MM-GBSA) Calculations, and Molecular Dynamics Simulations (MDS) to provide biophysical profiles of the conformational pattern of *Cannabis sativa* L. as Epidermal growth factor receptor tyrosine kinase inhibitors (EGFR TKIs). In this step of screening, we will consider the expected interaction patterns of the lead flexible ligands against the flexible active amino acid side chain in the EGFR-TKD active pocket, estimated binding energies (BE) of the ligands towards the receptor, estimate the micromolar (μM) values of the inhibitory concentrations (Ki), examine the structural and dynamic behavior of the proposed EGFR-TKIs.

• Predict binding energies and inhibitory activity concentrations

For this purpose, using a Lamarckian genetic algorithm (LGA) and an empirical binding free energy function available in AutoDock 4.2.6, we can get: The top conformational binding mode profiles (lowest free binding energy with the minimum RMSD distance cutoff) in all selected EGFR-inhibitors complexes. Also, estimate the inhibitory concentration required to yield 50% of the maximum inhibition of the EGFR-TKD enzymatic activity based on the theoretical inhibition constants ($Ki=e^{\frac{BE}{R\times T}}$, where R = 1.985 × 10^−3^ kcal K^−1^ × mol^−1^ and T = 298.15 K), the binding energy (BE) is the estimated free energy of binding, R is the ideal gas constant and T is the ambient temperature (298.15 K). (For details, see the supplementary information).

• MM-GBSA Free Energies (ΔG_bind_) assessments

Using the Prime/MM-GBSA computations provided in Schrodinger Suite 2020-3, at pH 7 ± 2 the free binding energy ΔG_bind_ for EGFR-TKIs systems were prepared according to the OPLS3e force field and the VSGB 2.1 solvent model [90,91]. This procedure was implemented as an aid protocol for molecular docking simulations towards the identification of potentially most stable systems for their validation by molecular dynamics simulation (for details, see the supplementary information). The examined EGFR-TKIs complexes were prepared and primed for Prime MM-GBSA scores using the protein prepwizard (Protein Preparation Wizard) on the Maestro 12.5 interface [92].

As part of the preparation, hydrogen was added to the 3D structure of the systems, disulfide bonds were formed, generated ionization/tautomeric states for all moieties (amino acid side chains and ligands), H-bond and system-based functional enhancement were optimized using OPLS3e (Optimized Potentials for Liquid Simulations) force field.

The Epik (Empirical pKa Prediction) function was incorporated during system preparation to provide appropriate resolution for protonation states of ligands during the drug discovery steps [93]. This was due to the pKa of the functional groups plays a major role in defining the pharmacokinetic profile and structural dynamics behavior of the functional groups that characterize the drug [93]. Epik module allowed us to reliably predict the protonated state of ligands, allowing us to understand the discrete interactions that cause ligands to bind to the target protein. However, in the virtual screening, we find a set of tools that provide known rigid structures containing all functional groups but do not take into account the protonation and tautomeric states of the ligands.

• Molecular dynamics simulations

Using Desmond/GPU, multiple 200 ns molecular dynamics simulations were performed to analyze the dynamic and structural behavior of refined samples of EGFR-inhibitor systems under the OPLS3e force field and in a water-based SPC (Simple Point-Charge) model (for details, see the supplementary information). This simulation aided in the final stage of the overall learning process of the stability dynamics of free EGFR and EGFR-TKIs complexes in a laboratory-like virtual model. The most negative free binding energies of the ligands with EGFR, the estimated Ki concentrations, and the most stable conformations of the ligands in terms of functional groups used to select protein-drug samples for molecular dynamics tests.

## 3 Result and discussion

### 3.1 Validation of the structural model of EGFR-TKD protein (PDB ID:1M17)

**Fig. 2:**
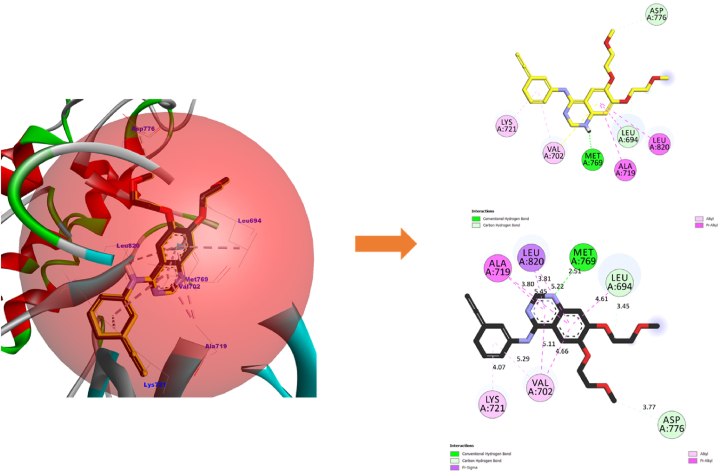
The conformational positions of the original Erlotinib inhibitor (yellow)/re-docked (black) inside the active EGFR-TKD pocket. (For interpretation of the references to color in this figure legend, the reader is referred to the Web version of this article.)

Fig. 2 shows a 3D/2D overview of the conformation of the native (yellow) and re-docked (black) Erlotinib drug in the EGFR-TKD protein active pocket. Results of the molecular re-docking simulation showed that the binding affinity of Erlotinib towards EGFR-TKD was −8.2 kcal/mol and the ideal RMSD value of the conformational superimposition mode b/w the ligands (original/re-docked) was 0.155 Å. It was also observed that the re-docked Erlotinib interacted with the identical amino acid residues as the original Erlotinib in the 1M17 crystal complex. The RMSD value less than 2 Å and the close proximity between the interaction profiles of the original and re-docked Erlotinib validate the molecular docking protocol used in the present investigation. Therefore, the rest of the molecular docking simulations in this study can be completed with confidence.

### 3.2 Screening the affinity of the ligands towards the active binding site

Table S2 reports the binding affinities of the ligands (*Cannabinoids Isolated*: C1–C12 and *Cannabis Terpenes*: T1-T38, Drugs: Tamoxifen, and Erlotinib) towards EGFR-TKD (PDB ID: 1M17). The results are presented in Table S2; which is compiled from the output of a semi-flexible molecular docking model performed by Autodock vina 1.1.2. From Table S2, we can notice that all values of binding affinities for docked ligands inside the active pocket of EGFR-TKD were significantly low (more negative). This indicates that *Cannabis sativa L*-based compounds are structurally consistent with EGFR-TKD, and that these compounds can establish a non-covalent binding with EGFR-TKD. Thus, these phytochemicals may be attractive reversible inhibitors of EGFR.

From Table S1, the binding affinities of ligands belonging to the *isolated cannabinoids* class recorded the highest value for **C2** (−7.2 kcal/mol) and the lowest value for **C11** (−11.3 kcal/mol). The ligands **C1** (−9.9 kcal/mol), **C4** (−9.8 kcal/mol), **C5** (−9.9 kcal/mol), **C6** (−10.8 kcal/mol), **C7** (−10.7 kcal/mol), **C8** (−10.1 kcal/mol), **C9** (−10.0 kcal/mol), **C10** (−8.4 kcal/mol) and **C11** (−11.3 kcal/mol) recorded more negative binding affinities than **Tamoxifen** (−9.4 kcal/mol) and **Erlotinib** (−8.2 kcal/mol), which means that the structural properties of these molecules are more selective and compatible with EGFR- TKD compared to the reference drugs Tamoxifen and Erlotinib. Therefore, **C1** (*Cannabidiolic acid*), **C4** (*Cannabidiol*), **C5** (*Tetrahydrocannabivarin*), **C6** (*Cannabinol*), **C7** (*Dronabinol*), **C8** (*Delta-8-Tetrahydrocannabinol*), **C9** (*Cannabicyclol*), and**C11** (*Delta9-tetrahydrocannabinolic acid*) phytochemicals can be screened as potential anticancer reversible inhibitors against breast and lung cancers. Whereas the binding affinities of the ligands (**C10** and **C12**) were less negative than those of **Tamoxifen** and more negative than those of **Erlotinib**, this means that both phytochemicals (**C10**: *Cannabichromene* and **C12***: Cannabichromenic Acid*) have promising prospects against lung cancer.

On the other hand, from Table S1 we can notice that the binding affinities of ligands belonging to the *Cannabis Terpenes* class reached the highest value −5.5 kcal/mol for **T2**, **T7** and **T11**, and the lowest value was −8.8 kcal/mol for **T17**. Apparently, all ligands (**T1**-**T38**) had a more limited negative binding affinities compared to **Tamoxifen**. This means that the interactions of the *Cannabis Terpenes* with EGFR-TKD are less likely to be compatible than those established by **Tamoxifen** and *Cannabinoids isolated* against breast cancer. However, the ligands **T15** (−8.4 kcal/mol), **T17** (−8.8 kcal/mol), **T25** (−8.4 kcal/mol), and **T28** (−8.5 kcal/mol) recorded more negative binding affinities than **Erlotinib** (−8.2 kcal/mol) toward the active EGFR-TKD pocket. This indicates that **T15** (Beta-Caryophyllene), **T17** (Nerolidol), **T25** (*Gamma-Elemene*), and **T28** (*Beta-Eudesmene*) are candidate reversible inhibitors agents for EGFR-TKD against lung cancer. Also, Table 1 shows that **Tamoxifen** has a higher negative binding energy towards EGFR (−9.4 kcal/mol) than **Erlotinib** (−8.2 kcal/mol), which may support its potential as a tyrosine kinase inhibitor that could specifically target EGFR. Therefore, repositioning **Tamoxifen** as a dual therapeutic strategy in lung and breast cancer targeted therapy may be effective, but further study and predictions are required. Table 1 summarizes filtered ligands based on their binding affinities and selectivity against breast and lung cancer.

**Table 1:** Overview of the binding affinity of candidate EGFR-TKD inhibitors.

Category of lead compound	Ligand name/2D Structure	binding affinity (kcal/mol)	Favorable affinity against
Isolated cannabinoids	C1 (**CBDA**)	−9.9	breast and lung

	C4 (**CBD**)	−9.8	breast and lung

	C5 (**THCV**)	−9.9	breast and lung

	C6 (**CNB**)	−10.8	breast and lung

	C7 (**Δ-9-THC**)	−10.7	breast and lung

	C8 (**Δ-8-THC**)	−10.1	breast and lung

	C9 (**CBL**)	−10.0	breast and lung

	C10 (**CBC**)	−8.4	lung

	C11 (**THCA**)	−11.3	breast and lung

	C12 (**CBCA**)	−8.5	lung

Cannabis Terpenes	T15 (**BCP**)	−8.4	breast and lung

	T17 (**NRD**)	−8.8	breast and lung

	T25 (**γ-Ele**)	−8.4	breast and lung

	T28 (**β-EUD**)	−8.5	lung

Standard drug	Tamoxifen	−9.4	breast

	Erlotinib	−8.2	lung

### 3.3 Drug-like properties and toxicity risks

Table 2 provides a profile of the drug-like properties and potential toxicity risks of the *Cannabis sativa* L. phytochemicals proposed in this study. Concerning the bioavailability parameters estimated on the bases of Lipinski RO5 and Veber's Rule, we can notice that the identified *Cannabis sativa* L. compounds satisfy all conditions necessary for a good bioavailability. This can be confirmed by the absence of violations in Lipinski RO5 and Vebers' criterion. For standard drugs, Tamoxifen and Erlotinib, respectively, there were two violations in lipophilicity (LogP>5) and number of rotational bonds (nROB = 10 bonds). This means that the structural properties of these two drugs may be insufficient to ensure their proper absorption by the human intestine, which may cause a poor response of the drug as desired.

**Table 2:** The Drug-like pattern and Toxicity risks of the screened molecules.

Comp.	Bioavailability criteria						DruglikenessVio.		F	SA	Tox-R
	MW	LogP	nHBA	nHBD	nROB	MR	RO5	Viber	>10%	1–10	+/±/-
	≤500 D	<5	<10	<5	<10	40–130	0 ≤ Vio≤2				
C1 (CBDA)	358.47	3.79	4	3	7	106.81	0	0	0.56	4.25	**-**
C4 (CBD)	314.46	4.31	2	2	6	99.85	0	0	0.55	4.05	**-**
C5 (THCV)	286.41	3.94	2	1	2	88.30	0	0	0.55	4.05	**-**
C6 (CNB)	310.43	4.23	2	1	4	97.10	0	0	0.55	3.39	**-**
C7 (Δ-9-THC)	314.46	4.39	2	1	4	97.91	0	0	0.55	4.27	**-**
C8 (Δ-8-THC)	314.46	4.39	2	1	4	97.91	0	0	0.55	4.21	**-**
C9 (CBL)	314.46	4.48	2	1	4	96.01	0	0	0.55	4.37	**-**
C10 (CBC)	314.46	4.31	2	1	7	100.34	0	0	0.55	4.26	+RE, +MG
C11 (THCA)	358.47	3.88	4	2	5	104.87	0	0	0.85	4.43	**-**
C12 (CBCA)	358.47	3.79	4	2	8	107.30	0	0	0.85	4.40	+RE, +MG
T15 (BCP)	204.35	4.63	0	0	0	68.78	0	0	0.55	4.51	**-**
T17 (NRD)	222.37	3.86	1	1	7	74.00	0	0	0.55	3.53	+IRR
T25 (γ-Ele)	204.35	4.53	0	0	2	70.42	0	0	0.55	3.81	**-**
T28 (β-EUD)	204.35	4.63	0	0	1	68.78	0	0	0.55	3.42	**-**
Tam	371.51	5.10	2	0	8	119.72	1	0	0.55	3.01	+RE
Erlo	393.44	1.89	6	1	10	111.40	1	0	0.55	3.19	**-**

**MW**: Molecular weight; **MR**: Molar Refractivity, **LogP**: Lipophilicity; **nHBA**: Num. H-bond acceptors; **nHBD**: Num. H-bond donors; **nROB**: Num. rotatable bonds; **MR**: Molar Refractivity, **nVio**: Num. volations, **F**: Bioavailability Score, **SA**: Synthetic accessibility.**Tox-R**: Potential toxicity risks: **IRR**: Irritant, **RE**:Reproductive effective, **MG**: Mutagenic, **TUMO**: Tumorigenic **(+:** highly toxic; **-:** not toxic; **±:** slightly toxic. **Tam**: Tamoxifen, **Erl**: Erlotinib.

Regarding the bioavailability score (F), values of F> 50% indicates that more than 50% of the orally dosed drug can reach the systemic circulation and allow them to cross a variety of barriers in the process of drug metabolism. Regarding the synthetic accessibility index (SA), values of SA <10 for the examined compounds indicate the easy synthesis and evaluation of these compounds *in vitro*. Regarding the toxicity risk (Tox-R) parameters evaluated by Osiris calculations, we can notice that the structures of the molecules **C10 (**CBC**)**, **C12** (CBCA), **C17** (NRD) and the reference drug **Tamoxifen** can cause toxicity risks represented by the influence on the reproduction, mutagenicity and irritation when these molecules are administered as a drug.

The overall drug-like predictions, combined with Osiris calculations, indicate that the phytochemical structures **C1** (CBDA), **C4** (CBD), **C5** (THCV), **C6** (CNB), **C7** (Δ-9-THC), **C8** (Δ-8-THC), **C9** (CBL), **C11** (THCA), **T15** (BCP), **T25** (γ-Ele)**,** and **T28** (β-EUD) screened from *Cannabis sativa* L. may be more compatible and suitable for use as a better anti-cancer drug compared to the reference drugs **Tamoxifen** and **Erlotinib**. Therefore, to avoid these risks, we exclude the phytochemicals **C10** (CBC), **C12** (CBCA) and **T17** (NRD) from safe drug candidate selection. Consequently, we analyze *in silico* the ADME-Tox properties of the remaining phytochemicals **C1** (CBDA), **C4** (CBD), **C5** (THCV), **C6** (CNB), **C7** (Δ-9-THC), **C8** (Δ-8-THC), **C9** (CBL), **C11** (THCA), **T15** (BCP), **T25** (γ-Ele), and **T28** (β-EUD).

### 3.4 *In silico* ADME-tox modeling

For our selected set of phytochemicals cannabinoids **C1** (CBDA), **C4** (CBD), **C5** (THCV), **C6** (CNB), **C7** (Δ-9-THC), **C8** (Δ-8-THC), **C9** (CBL), **C11** (THCA) and the cannabis terpenes **T15** (BCP), **T25** (γ-Ele), **T28** (β-EUD), we have noticed that majority of compounds represented highest % human oral absorption (HOA) profiles (>90%), when calculated using ‘ADME-Tox’ (Table S3). Our best docked compound, **C11** (>97.56% HOA) retained an absorption comparable to standard drugs, Tamoxifen (Tam) and Erlotinib (Erlo), i.e., Erlo (97.80% and 95.26% HOA, respectively). Furthermore, isolated cannabinoids (**CBDA**, **CBD**, **THCV**, **CNB**, **Δ-9-THC**, **Δ-8-THC**, **CBL**, **THCA**) demonstrated comparable skin permeability (log Kp) with respect to drugs, Tamoxifen and Erlotinib (log Kp: 2.73 each).

Among isolated cannabinoids, **Δ-9-THC**, **Δ-8-THC**, **CBL** are likely to exert lesser pharmacological actions, as denoted by little to no unbound fractions. Consequently, it is possible that these compounds have a structure appropriate for binding to the protein and not to the plasma. However, it is worthy to note that, cannabis terpenes, **BCP** and **γ-Ele** had noted with significant unbound fractions (0.26 and 0.14, respectively) comparable to drugs (Tam: 0.16 and Erl: 0.14). The steady state volume of distribution (VDss) and the blood-brain barrier (BBB) permeability properties were also found to be optimum for compounds in current study (Table S3). The total body clearance or total plasma (blood) clearance (CL) is commonly defined as the volume of plasma (blood) completely cleared of drug per unit time [94].

With this aspect, isolated cannabinoids and cannabis terpene showed to have high total clearance rates. Except compounds, **CNB** and **β-EUD**, all compounds were found to have no ‘AMES toxic’ profile. If a species has a high ORAT LD_50_ it means it has a high tolerance to the poison. A low ORAT LD_50_ means the species is highly susceptible to the poison. Considering this analogy, we noticed that except compounds, **BCP**, **γ-Ele**, and **β-EUD**, all others, isolated cannabinoids exhibited higher LD_50_ values than drug, Tamoxifen, but lesser than Erlotinib. Phytochemicals (**CBDA**, **CBD**, **THCV**, **CNB**, **Δ-9-THC**, **Δ-8-THC**, **CBL**, **THCA**) and (**BCP**, **γ-Ele** and **β-EUD**) depicted no hepatotoxicity. It is well known that Cytochrome P450 (CYP450) enzyme family plays crucial roles in metabolism of drug/xenobiosis. Substrate and inhibitory profiles for particular cytochrome enzyme, may lead alteration in drug metabolism and thus, generation of metabolites, which may alter pharmacological actions or excretion of drug. Phytochemicals (**CBDA**, **CBD**, **THCV**, **CNB**, **Δ-9-THC**, **Δ-8-THC**, **CBL**, **THCA**) and (**BCP**, **γ-Ele** and **β-EUD**) represented no substrate or inhibitory profiles against CYP-2D6 enzyme. Compounds, **CBD**, **CNB, CBL**, and **β-EUD**, including standards, found to be substrate for CYP-3A4. Further, except **Δ-9-THC** and **Δ-8-THC**, all others depicted non-inhibitory profile for CYP-3A4.

Phytochemicals, **THCV**, **CNB**, **Δ-9-THC**, **Δ-8-THC**, **CBL** exhibited inhibition against CYP-2C19. Among all phytochemicals, only **C6** (**CNB**) would likely to show CYP-2C9 inhibition. All of these pharmacokinetics (PK) and pharmacodynamics (PD) properties are summarized in Table S3. Based on evaluation of the PK/PD properties (ADME-Tox) of the investigated phytochemicals as EGFR-TKIs, C1 (**CBDA**), C4 (**CBD**), C5 (**THCV**), C7 (**Δ-9-THC**), C8 (**Δ-8-THC**), and C9 (**CBL**), C11 (**THCA**), T15 (**BCP**), and T28 (**γ-ELE**) all have properties favorable for safe drug use, except for C6 (**CNB**) and T28 (**β-EUD**), which show potential toxicity. In this regard, an *in vitro* study conducted by Lamtha et al. [50] suggested that cannabinoid molecules **CBD**, **CBG** and **CBN** could act as EGFR-TKIs. However, their study did not assess the PK and PD properties of these molecules. In the present study, **CNB** was excluded from the drug candidates due to its potential toxicity, whereas **CBD** showed drug-like and ADME-Tox properties favorable for its use as a drug. On the other hand, **CBG** was not considered a suitable EGFR-TKI in this study due to its lower protein affinity (−7.2 kcal/mol) compared to the two standard drugs Erlotinib (−8.2 kcal/mol) and Tamoxifen (−9.4 kcal/mol).

### 3.5 Conformational stability analysis of EGFR- TKIs complexes

#### 3.5.1 Characterization of screened EGFR-TKIs

Table S4 depicts the main results of the flexible molecular docking performed by Autodock 4.2.6 as well as the Prime MM-GBSA computations. The flexible molecular docking results generated the following parameters: estimated binding free energy (BE), estimated biological inhibitory activity (Ki), and interaction patterns between the ligands and the active site of EGFR-TKD.

Whereas, ΔG_bind_ for EGFR-inhibitor complexes were generated from Prime/MM-GBSA calculations. The interactions of the proposed EGFR-TKIs (**C1**, **C4**, **C5**, **C7**, **C8**, **C9**, **C11**, **T15**, and **T25**) with the active EGFR-TKD pocket in the presence of **Tamoxifen** and **Erlotinib** are shown in Figs. S1 and S2, respectively, as predicted by flexible molecular docking and Prime MM-GBSA simulations.

The detailed findings described in Table S4 and Figs. S1 and S2 lead us to the following conclusions: The binding energies (BE) estimated by molecular docking ranged from −12.79 kcal/mol (**EGFR-C11** complex) to −8.16 kcal/mol (**EGFR-C4** complex) for the lead compounds belonging to isolated cannabinoids, and from −7.61 kcal/mol (**EGFR-T15** complex) to −7.41 kcal/mol (**EGFR-T25** complex) for the lead compounds belonging to cannabis terpenes. In comparison, the binding energy values of the two reference drugs ranged from −8.79 (**EGFR-Tamoxifen** complex) to −8.29 kcal/mol (**EGFR-Erlotinib** complex).

Inhibitory concentration (Ki) values were estimated by the flexible molecular docking ranged from 0.4115 E^−3^ μM (**EGFR-C11** complex) to 1.0281 μM (**EGFR-C4** complex) for lead compounds belonging to isolated cannabinoids, and from 2.603 μM (**EGFR-T15** complex) to 3.650 μM (**EGFR-T25** complex) for lead compounds belonging to cannabis terpenes. In comparison, the estimated inhibitory concentration values for the reference drugs were ranged from 0.4000 μM (**EGFR-Tamoxifen** complex) to 0.8253 μM (**EGFR-Erlotinib** complex).

The free binding energies (ΔG_bind_) were set to limits by Prime MM-GBSA module and ranged from −62.807 kcal/mol (**EGFR-C11** complex) to −31.138 kcal/mol (**EGFR-C1** complex) for isolated cannabinoids, and from −39.782 kcal/mol (**EGFR-T15** complex) to −33.980 kcal/mol (**EGFR-T25** complex) for cannabis terpenes. The binding energy values of the two standard drugs were ranged from −42.098 (**EGFR-Tamoxifen** complex) to −36.877 kcal/mol (**EGFR-Erlotinib** complex).

In all isolated cannabinoid complexes, the protein-ligand interactions were found to have hydrogen bond (conventional/carbon), hydrophobic, electrostatic (**EGFR-C11** complex), and sulfur (**EGFR-C5** and **EGFR-C7** complexes), whereas in cannabis terpene complexes (**EGFR-T15** and **EGFR-T25**), all interactions were of hydrophobic (Alkyl). Tamoxifen was interacted with the active amino acid residues in the EGFR pocket via hydrogen carbon bonds, hydrophobic interactions, and electrostatic (π-Cation) interactions, whereas Erlotinib interactions were hydrogen bond (conventional and carbon) and hydrophobic interactions.

Furthermore, in the inhibition of EGFR-TKD enzymatic activity, isolated cannabinoids and cannabis terpenes were able to generate multiple interactions in different modes with a set of reference amino acid residues. Visualizations of interacting candidate drug molecules with EGFR, are shown in Fig. S1. Following the MM-GBSA simulation, all of the examined ligands were not diverged from the active pocket of EGFR-TKD (Fig. S2), indicating that the proposed drug molecules' structure could achieve perfect conformance with the EGFR-protein TKD's structure.

Predictions of Epik (Empirical pKa Prediction) function under simulated physiological conditions were also indicated that the carboxylic acid moiety (COOH) in the **C1** (CBDA) and **C11** (THCA) structures was ionized to a carboxylate ion by loss of $H^{+}$, whereas the other functional moieties of the ligands **C4** (CBD), **C5** (THCV), **C7 (**Δ-9-THC**)**, **C8 (**Δ-8-THC**)**, **C9** (CBL), **T15** (Δ-8-THC), **T25** (γ-Ele) and **Erlotinib** were not protonated. Whereas **Tamoxifen** was ionized the ammonia (${\text{NH}}_{3}$) moiety by the receipt of the $H^{+}$ proton which formed the ammonium cation (${\text{NH}}_{4}^{+}$) on the **Tamoxifen** structure.

#### 3.5.2 Validation of protein-ligand interactions' stability

In comparison to the two standard drugs (**Tamoxifen** and **Erlotinib**), overall results of the rational screening on *Cannabis sativa* L. showed that phytochemicals (**CBDA**, **CBD**, **THCV**, **Δ-9-THC**, **Δ-8-THC**, **Δ-8-THC**, **THCA**, **Δ-8-THC**, and **γ-Ele**) had flexible structural properties and favorable PK/PD properties to be used as safe cancer drugs. Because of their small molecular structures, these phytochemicals can bind to the active pocket of EGFR-TKD and perform several non-covalent interactions that are beneficial in inhibiting EGFR-TKD enzymatic activity. Therefore, there is great hope that these compounds will generate a new generation of EGFR-TKIs for targeted cancer therapy.

**Fig. 3:**
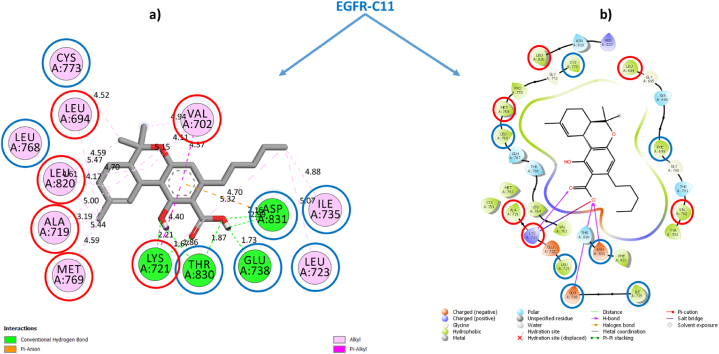
EGFR-C11 interactions. **(a)** Flexible molecular docking predictions. **(b)** Prime MM-GBSA predictions. Amino acids surrounded by the red circle indicate reference sites, amino acids surrounded by the blue circle indicate novel active sites. (For interpretation of the references to color in this figure legend, the reader is referred to the Web version of this article.)

To validate this conclusion, we selected one sample from each category of Cannabinoids and Terpenes and closely examine their structural stabilities in situ inside the EGFR-TKD active pocket. Within each category, we have taken the sample with the best estimated inhibitory concentration (low Ki) and the lowest free binding energy estimated via Prime MM-GBSA computations as the samples for further examination. To this end, we had selected the phytochemical **THCA** (Ki = 0.4115 × 10^−3^ μM, ΔG_bind_ = −62.807 kcal/mol) as a representative sample standard for the isolated cannabinoids category and the phytochemical **BCP** (Ki = 2.603 μM, ΔG_bind_ = −33.980 kcal/mol) as a representative standard for the cannabis terpenes category and **Tamoxifen** (Ki = 0.400 μM), ΔG_bind_ = −42.098 kcal/mol) and **Erlotinib** (Ki = 0.8253 μM, ΔG_bind_ = −36.877 kcal/mol) as standarddrugs. Table 3 shows the most important parameters related to the interactions of the reference active amino acid residues (**Leu694**, **Val702**, **Ala719**, **Lys721**, **Met769**, **Asp776**, and **Leu820**) in the EGFR-TKD pocket with the samples **C11** (**THCA**), **T15** (**BCP**), **Tamoxifen**, and **Erlotinib**. Fig. 3 depicts a 2D visualization of the conformational profile of the selected samples' interactions in the EGFR-TKD active pocket (**EGFR-C11**, **EGFR-T15**, **EGFR-Tamoxifen** and **EGFR-Erlotinib**).

**Table 3:** Interactions of sample TKIs with EGFR.

Samples		EGFR-TKIs non-covalent Interactions			Ki (μM)	ΔGbind (kcal/mol)
		Hydrogen Bond	Hydrophobic	Electrostatic		
THCA	EGFR-C11	Conventional 7: THR830 (1.86, 1.85 Å), ASP831 (2.16, 2.99 Å), GLU738 (1.73 Å), LYS721 (2.21, 1.66 Å). Carbon 0.	Alkyl 16: ALA719 (4.99, 3.18 Å), MET769 (5.44, 4.60 Å), LEU820 (4.17, 4.60 Å), CYS773 (4.52 Å), VAL702 (4.94, 4.10, 5.15 Å), VAL768 (4.60 Å), LEU694 (5.46 Å), LEU723 (5.06 Å), ILE735 (4.88 Å), LYS721 (5.32 Å), LEU820 (4.70 Å). π-Alkyl 2: VAL702 (4.37 Å), LYS721 (4.40 Å). π-π T-shaped 0.π-Sigma 0	π-Anion 1: ASP831 (4.70 Å)	0.4115 × 10-3	−62.807
BCP	EGFR-T15	ND	Alkyl 16: ALA719 (3.72, 3.99, 4.81 Å), VAL702 (4.77, 4.60, 3.75, 4.18 Å), LYS721 (5.32, 4.74, 4.07), MET769 (4.92, 4.45 Å), LEU820 (4.96, 4.92 Å), LEU694 (5.29 Å).	ND	2.603	−33.980
Standard drugs	EGFR-Tamoxifen	Carbon 1: ASN818 (2.93 Å)	π-Sigma 1: ALA 719 (3.73 Å). Alkyl 5: ALA719 (4.11 Å), LEU694 (4.83 Å), VAL702 (5.22 Å), MET769 (4.30 Å), LEU820 (4.84 Å) π-Alkyl 6: VAL702 (4.98, 4.58, 5.23 Å), LYS721 (4.98, 4.02 Å), LEU820 (4.89 Å). π-π T-shaped 1: PHE699 (5.13 Å).	π-Cation 2: LYS721 (4.26, 4.96 Å)	0.400	−42.098
	EGFR-Erlotinib	Conventional 4: MET769 (2.65 Å), LYS721 (1.78, 2.22, 2.06 Å). Carbon 2: ASP831 (3.51 Å), GLU738 (2.83 Å).	π-Sigma 1: 1: VAL702 (3.91 Å). π-Alkyl 9: LEU694 (5.38, 4.17, 5.45 Å), ALA719 (5.24, 4.88 Å), LEU768 (5.41 Å), LEU769 (5.06 Å), MET769 Å (4.82 Å), LEU820 (5.14 Å)	ND	0.825	−36.877
Reference flexible amino acid residues		Leu694, Val702, Ala719, Lys721, Met769, Asp776 and Leu820				

ND: Not detected. •EGFR-THCA interactions

**Fig. 4:**
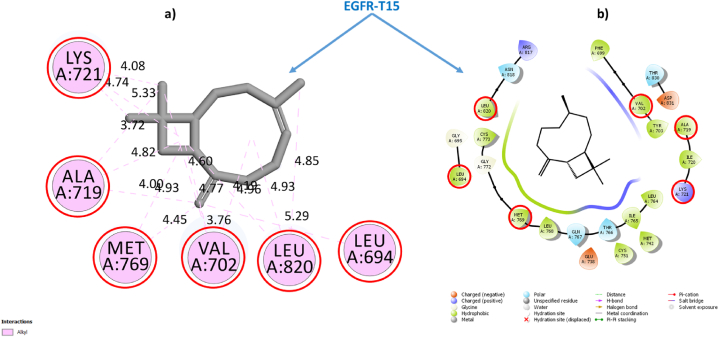
EGFR-T15 interactions. **(a)** Flexible molecular docking predictions. **(b)** Prime MM-GBSA predictions. Amino acids surrounded by the red circle indicate reference sites, amino acids surrounded by the blue circle indicate novel active sites. (For interpretation of the references to color in this figure legend, the reader is referred to the Web version of this article.)

**Fig. 5:**
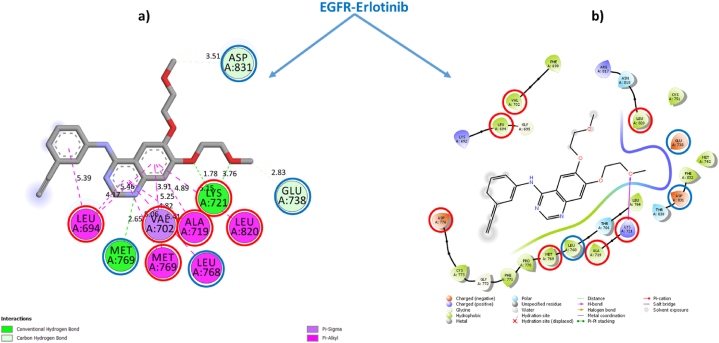
EGFR-Erlotinib interactions. **(a)** Flexible molecular docking predictions. **(b)** Prime MM-GBSA predictions. Amino acids surrounded by the red circle indicate reference sites, amino acids surrounded by the blue circle indicate novel active sites. (For interpretation of the references to color in this figure legend, the reader is referred to the Web version of this article.)

**Fig. 6:**
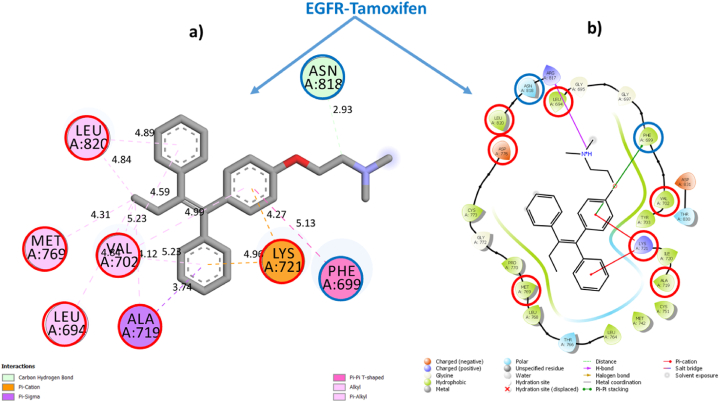
EGFR-Tamoxifen interactions. **(a)** Flexible molecular docking predictions. **(b)** Prime MM-GBSA predictions. Amino acids surrounded by the red circle indicate reference sites, amino acids surrounded by the blue circle indicate novel active sites. (For interpretation of the references to color in this figure legend, the reader is referred to the Web version of this article.)

Fig. 3, Fig. 4, Fig. 5, Fig. 6 depict ligand interactions in the EGFR active pocket obtained through flexible molecular docking, while Fig. 3, Fig. 4, Fig. 5, Fig. 6 depict ligand interactions in the EGFR active pocket obtained through Prime MM-GBSA calculations in the VSGB 2.1 solvation model. For reference, Schrodinger algorithms change the names of standard residues such as **ARG**, **ASP**, **GLU**, **LYS**, and **HIS** to nonstandard residues **RNA**, **ASH**, **GLH**, **LYN**, **HIE**, and **HIP** as an informal convention for assigning different proton states to regular residues.

For the EGFR-C11 complex, flexible molecular docking simulations were indicated that the binding of the C11 (**THCA**) to the EGFR active pocket was achieved through the formation of non-covalent interactions of conventional hydrogen bonds with residues THR830 (1.86, 1.85 Å), ASP831 (2.16, 2.99 Å), GLU738 (1.73 Å), LYS721 (2.21, 1.66 Å). In addition, it also formed hydrophobic alkyl interactions with residues ALA719 (4.99, 3.18 Å), MET769 (5.44, 4.60 Å), LEU820 (4.17, 4.60 Å), CYS773 (4.52 Å), VAL702 (4.94, 4.10, 5.15 Å), VAL768 (4.60 Å), LEU694 (5.46 Å), LEU723 (5.06 Å), ILE735 (4.88 Å), LYS721 (5.32 Å), LEU820 (4.70 Å). π-Alkyl 2: VAL702 (4.37 Å), LYS721 (4.40 Å) and π-Alkyl interactions with VAL702 (4.37 Å), LYS721 (4.40 Å), and interacted with residue ASP831 (4.70 Å) by the π-Anion electrostatic bonding. Among these interactions, the optimized **THCA** structure successfully formed multiple bonds with six side chain residues of the reference amino acid in the inhibition of EGFR enzyme activity, specifically LEU694, LEU820, ALA719, MET769, LYS721 and VAL702.

In addition, **THCA** interacted with seven novel active residues in the EGFR pocket, namely CYS773, LEU768, THR830, GLU738, ASP831, LEU723 and ILE735. On the other hand, the predictions of the Prime MM-GBSA energy calculations in VSGB solution indicated the stability of the **THCA** molecule structure within the EGFR active pocket and the maintenance of the same predicted interactions due to flexible molecular docking. This confirms that the **THCA** sample may be suitable for inhibiting EGFR enzymatic activity as well as achieving stability and compatibility with it in order to achieve the therapeutic goal. This means that the phytochemical compounds (**CBDA**, **CBD**, **THCV**, **Δ-9-THC**, **Δ-8-THC, CBL** and **THCA**) of the isolated cannabinoids may be potent new anticancer candidates based on targeting EGFR-TKD enzymatic activity.

• EGFR-BCP interactions

For the EGFR-**T15** complex, flexible molecular docking indicated that the interaction of **BCP** with the active EGFR pocket was done through the generation of non-covalent hydrophobic alkyl-like interactions with residues ALA719 (3.72, 3.99, 4.81 Å), VAL702 (4.77, 4.60, 3.75, 4.18 Å), LYS721 (5.32, 4.74, 4.07), MET769 (4.92, 4.45 Å), LEU820 (4.96, 4.92 Å), LEU694 (5.29 Å).

All amino acid residues with which **T15** interacts in the EGFR active pocket are reference in the inhibition of EGFR-TKD enzymatic activity. Prime MM-GBSA simulations also indicated the stability of **T15** within the active site in the EGFR pocket, confirming that the conformational structure of **BCP** is more suitable for inhibiting EGFR-TKD enzymatic activity. This suggests that cannabis terpene phytochemicals (**BCP** and **γ-Ele**) may be novel potent anticancer inhibitors of EGFR-TKD enzyme activity.

• EGFR-Erlotinib

For the EGFR-Erlotinib complex, flexible molecular docking indicated that binding of **Erlotinib** to the EGFR active pocket was via the generation of non-covalent interactions via conventional hydrogen bonds with residues MET769 (2, 65 Å), LYS721 (1.78, 2.22, 2.06 Å) and carbon hydrogen bonds with residues ASP831 (3.51 Å), GLU738 (2.83 Å), as well as via the formation of hydrophobic π-Sigma interactions with VAL702 (3.91 Å) and with residues LEU694 (5.38, 4.17, 5.45 Å), ALA719 (5.24, 4.88) Å), LEU768 (5.41 Å), LEU769 (5.06 Å), MET769 Å (4.82 Å), LEU820 (5.14 Å) via π-Alkyl interactions. The interaction of **Erlotinib** in the EGFR active pocket was observed to preserve six reference interactions with residues LEU694, VAL702, ALA719, LYS721, MET769, LEU694.

The non-interaction with ASP776 is due to the optimization of the Erlotinib structure and also took into account the resulting torsional energies on the free energy emitted due to the rotational bonds found in the flexible ligand especially for the Dimethoxyethane moiety, as well as the flexibility of the amino acid residues used as references in the virtual flexible molecular docking screening. We can notice that following flexible molecular docking of the reference drug **Erlotinib**, the reference drug conformation generated new bonds with ASP831, GLU738, LEU768 and MET769. The conservation of all interactions in the EGFR-Erlotinib complex was confirmed by Prime MM-GBSA computations, thus validating the EGFR-Erlotinib model (PDB ID: 1M17) as a reference to simulate the mechanisms of Protein-drug interactions via its targeting. Thus, **Erlotinib** can be used as a reference in rational and theoretical comparisons for lung cancer drug design and discovery.

• EGFR-Tamoxifen

For the **EGFR-Tamoxifen** complex, flexible molecular docking simulations indicated that **Tamoxifen** bound to the active EGFR pocket by generating non-covalent hydrogen-carbon bond interactions with ASN818 (2.93 Å), in addition it formed hydrophobic π-Sigma interactions with **ALA719** (3.73 Å), alkyl interactions with residues **ALA719** (4.11 Å), LEU694 (4.83 Å), **VAL702** (5.22 Å), **MET769** (4.30 Å), **LEU820** (4.84 Å), π-Alkyl interactions with residues **VAL702** (4.98, 4.58, 5.23 Å), **LYS721** (4.98, 4.02 Å), **LEU820** (4.89 Å), T-shaped π-π interactions with PHE699 (5.13 Å), and electrostatic π-Cation interactions with **LYS721** (4.26, 4.96 Å). Prime MM-GBSA simulations showed that **Tamoxifen** retained most of its predicted interactions by molecular docking in the EGFR-TKD active pocket. **Tamoxifen**'s interaction with the reference amino acid residues **ALA719**, **VAL702**, **MET769**, **LEU820**, and **LYS721** further supports its potential as an EGFR inhibitor. Therefore, the selection of this drug as a reference in inhibiting EGFR enzymatic activity against breast cancer is valid for rational comparison and theoretical screening.

### 3.6 Molecular dynamics analysis (MDA)

The results of the obtained screenings indicated that the structures of the phytochemicals selected from the categories Cannabinoids and Terpenes are in high accordance with the structure of EGFR-TKD. This gives these phytochemicals a potential chance to form a new generation of EGFR-TKD inhibitors and to reach therapeutic targets against lung and breast cancer. To further verify, we simulated the molecular dynamics in the aqueous environment of C11 (**THCA**) and T15 (**BCP**) samples in the presence of the reference drugs **Erlotinib** and **Tamoxifen**. Molecular dynamics simulations of EGFR-TKD and its filtered complexes (EGFR-C11, EGFR-T15, EGFR-Erlotinib, and EGFR-Tamoxifen) were carried out for 200 ns using the same approach described in **SI**.

The dynamical and structural properties of the EGFR-TKIs sample systems were analyzed in terms of time scales of Root Mean Square Deviation/Fluctuation(RMSD/RMSF)/ of atomic positions, Protein-ligand interactions, Ligand structural properties (ligand RMSD, Radius of Gyration, Intra-molecular Hydrogen Bonds, Molecular Surface Area, Solvent-Accessible Surface Area, and Polar Surface Area), and thermodynamic properties of the (Total Energy, Potential Energy, Temperature, Volume, and Pressure).

#### 3.6.1 RMSD and RMSF analysis

**Fig. 7:**
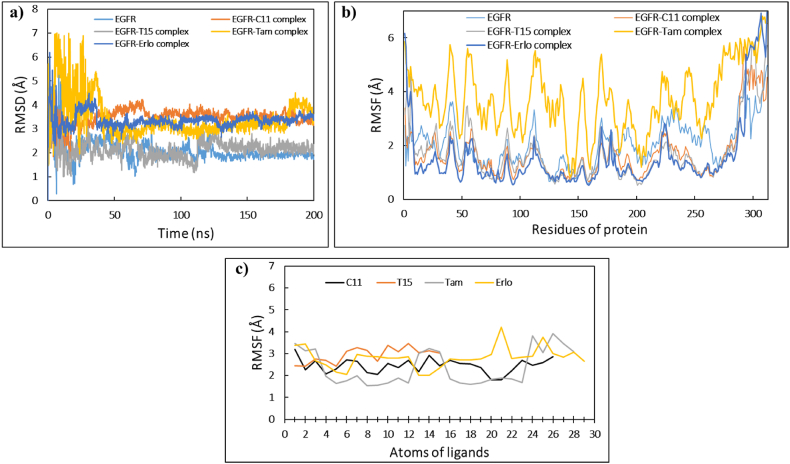
The MD simulations timelines for the EGFR systems uncomplexed, and complexed with the ligands C11, T15, Tamoxifen, and Erlotinib. (a) RMSD of C-alpha backbones of EGFR systems, (b) RMSF of side chains of EGFR systems, (c) RMSF of ligands complexed with EGFR.

From Fig. 7a, we can deduce that the RMSD values of the EGFR backbone systems stabilized after ∼40 ns of the MD simulations path ranging up to ∼200 ns. The average values of the RMSD±SD evolutions of the average thermal structure derived between the reference frame (t = 0 ns) and the final frame (t = 200 ns) for the free EGFR, EGFR-Tamoxifen, EGFR-Erlotinib, EGFR-C11, EGFR-T15 backbone systems, were 2.10 ± 0.45 Å, 3.48 ± 0.34 Å, 2.14 ± 0.37 Å, 3.44 ± 0.81 Å and 3.35 ± 0.30 Å, respectively. SD (standard deviation) values less than 1 Å indicate that the structural conformation of the free and complexed EGFR protein did not show significant conformational changes during the MD simulations. This indicates that the structure of the EGFR protein is in perfect fit and equilibrium with the lead drag ligands **THCA** and **BCP** as well as with the reference drugs **Tamoxifen** and **Erlotinib**.

The root means square fluctuations (RMSF) shown in Fig. 7b describe the local changes along the amino acid sequence of EGFR protein during the MD simulation run. The average values of RMSF±SD changes for the free EGFR and complexed with **THCA**, **BCP**, **Tamoxifen** and **Erlotinib** ligands were 2.02 ± 0.81 Å, 1.70 ± 0.95 Å, 1.72 ± 1.06 Å, 3.72 ± 1.22 Å, 1.58 ± 1.29 Å, respectively. It can be seen that the amino acid chain of the EGFR-Tamoxifen system underwent significant changes from the start to the end of the simulation, that can be explained by the significant average RMSF value of 3.72 Å. While the side chains of uncomplexed EGFR protein, EGFR-C11, EGFR-T15 and EGFR-Erlotinib, showed slight changes that can be explained by the average RMSF values ranging from 1.58 to 2.02 Å. It is also observed that the tails (*N*- and C-terminal) of the amino acid side chains fluctuate more than any other part of the protein, especially the amino acids numbered between 290 and 312 that are GLU961, ARG962, MET963, HIS964, LEU977, MET978, ASP9789, GLU978, GLU981, ASP982, MET983, ASP984, ASP985, VAL986, VAL987, ASP988, ALA989, ASP990, GLU991, TYR992, LEU993, ILE994, and PRO995.

Alignment of the protein-ligand complex to the uncomplexed protein backbone allows for the measurement of ligand-RMSF on the heavy atoms of the ligand. This provides insight into how the ligand fragments interact with the protein and bind in the active pocket of the protein. The ligand RMSF plot shown in Fig. 7c indicates the timeline of per-atom segmented ligand fluctuations, which corresponds to the two-dimensional structure of the C11 (**THCA**), T15 (**BCP**), **Tamoxifen**, and **Erlotinib** ligands.

The observed fluctuations of RMSF values for the ligands **THCA**, **BCP**, **Tamoxifen**, and **Erlotinib** reflect the fact that these ligands exhibit large internal atomic fluctuations during interaction with EGFR protein. This can be explained by the flexibility properties of this small molecule ligands. These properties allow the small molecules to provide various conformations and interaction patterns in the receptor protein cavity.

#### 3.6.2 Protein-ligand interactions

**Fig. 8:**
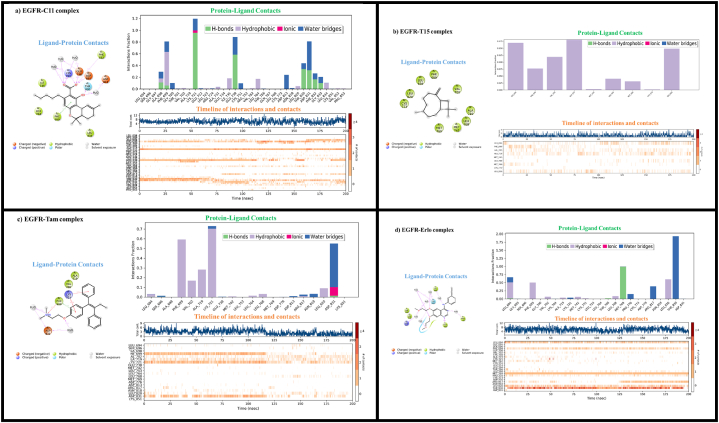
Detailed diagrams of local interaction dynamics of the ligands C11 (**THCA**), T15 (**BCP**), Tamoxifen and Erlotinib in the EGFR active pocket: (a) EGFR-C11 complex, (b) EGFR-T15 complex, (c) EGFR-Tamoxifen complex and (d) EGFR-Erlotinib.

Fig. 8 shows the profile of interactions that occur between the amino acid residues of EGFR with the ligands, C11 (**THCA**), T15 (**BCP**), Tamoxifen, and Erlotinib during the simulation time of the selected trajectory (0.00–200.00 ns). “Ligand-Protein Contacts” plots showing interactions that occur more than 10.0% of the simulation time between the atoms of the ligand and the amino acid residues of the protein. “Protein-ligand contacts” plots showing the time fractions of Protein-ligand interactions that maintained during the course of the simulation.

“Timeline of interactions and contacts” diagram depicts timelines of interactions and contacts (H-bonds, hydrophobic, ionic, water bridges), the top panel shows the total number of specific contacts to the protein with which the ligand interacts during the simulation time course. The bottom panel of the diagram gives in detail the residues that interact with the ligand in each frame of the MD simulation course. Residues with more than one contact with the ligand are represented by darker shades of orange, as scaled on the right of the diagram.

From Fig. 8a, we can notice that the interactions of ligand **THCA** with the active amino acid residues in EGFR-C11 complex are stabilized via H-bond, hydrophobic, ionic, water-bridge like interactions. The amino acid residues ALA698, PHE698, **PHE699**, GLY700, **VAL702**, **LYS721**, ALA731, ILE735, GLU738, **CYS751**, LEU764, ASP813, **LEU820**, **THR830**, ASP831, PHE832, GLY833, and LEU834 have been identified as the main contacts with which the ligand C11 maintained their interactions during the MD simulation.

According to Fig. 8b (EGFR-T15 complex), the stability of the ligand **BCP** with the EGFR protein is due to hydrophobic interactions with the amino acid residues **LEU694**, **PHE699**, **VAL702**, ALA719, **MET742**, **LEU768**, MET769, CYS773, and **LEU820**.

According to Fig. 8c (EGFR-Tam complex), the majority of Tamoxifen-EGFR contacts were hydrophobic interactions with residues **LEU694**, **PHE699**, **VAL702**, **ALA719**, **LYS721**, LEU764, **LEU768**, **LEU820**, as well as H-bonding, water bridging, and ionic interactions with ASP831.

According to Fig. 8d (EGFR-Erlo complex), water bridges and hydrophobic interactions with residues **LEU694**, **PHE699**, **VAL702**, **ALA719**, **LYS721**, **MET742**, **CYS751**, **LEU768**, PRO770, ARG817, **LEU820**, **THR830** as well as H-bond interactions with residues THR766 and MET769 are maintained between the Erlotinib drug and EGFR protein.

The residues LEU694, PHE699, VAL702, LYS721, CYS751, LEU820, THR830, LEU768, MET742 were identified as the major reference contacts with which the **THCA** and **BCP** ligands-maintained interactions during the MD simulation according to analysis of protein-ligand contact patterns using Tamoxifen and Erlotinib as references. As a result, the amino acid residues of Leucine, Phenylalanine, Valine, Lysine, Cysteine, and Threonine in the EGFR-TKD protein pocket can be considered as a key reference site favorable for threat modeling and achieving the desired biological function in order to inhibit the growth of breast and lung cancer cell lines by the phytochemical compounds of *Cannabis sativa* L (Cannabinoids and Terpenes).

#### 3.6.3 Ligands properties

**Fig. 9:**
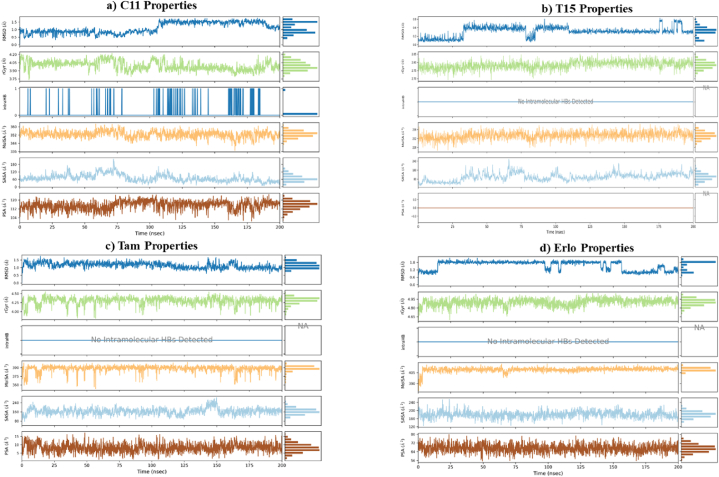
Plots of ligand properties variations in the EGFR active pocket throughout the simulation trajectory (0.00–200.00 nsec): (a) C11 ligand (THCA), (b) T15 ligand (BCP), (c) Tamoxifen drug, (d) Erlotinib drug.

Fig. 9(a-d) shows the time courses of the most important properties of the ligands C11 (**THCA**), T15 (**BCP**), Tamoxifen and Erlotinib recorded during the MD simulation. The ligand RMSD plot shows the variations in the ligand's root mean square deviation (from 0.00 to 200.00 nsec) with respect to the first reference conformation (time t = 0). The plot of radius of gyration (rGyr) variations indicates the ligand's expansion, and the rGyr parameter reflects the ligand's main moment of inertia in this plot. The intramolecular hydrogen bonding (intraHB) plot represents the time tracking of the number of internal hydrogen bonds (HB) detected inside the ligand. The molecular surface area plot (MolSA) represents the molecular surface area calculated with a probe radius of 1.4 Å; the MolSA parameter measurement is equivalent to the van der Waals surface area. The surface area of a molecule that can be reached by one molecule of water is expressed by the solvent accessible surface area (SASA) plot. The polar surface area (PSA) plot is used to calculate the surface area of a molecule accessible to the solvent, to which only the oxygen and nitrogen atoms contribute.

From Fig. 9(a-d), we can see that the RMSD property values for the ligands C11 (**THCA**), T15 (**BCP**), Tamoxifen, and Erlotinib stabilized within 0.5-1 Å (Fig. 9a), 0.3–0.9 Å (Fig. 9b), 1-1.5 Å (Fig. 9c), and 1-1.2 Å (Fig. 9d), respectively. For C11, T15, Tamoxifen, and Erlotinib ligands, respectively, the radius of gyration (rGyr) stability parameter ranged from 3.9-4 Å (Fig. 9a), 2.75–2.80 Å (Fig. 9b), 4.00–4.25 Å (Fig. 9c), and 4.80–4.95 Å (Fig. 9d). One intramolecular hydrogen bond (IntraHB) was found in the structure of the ligand **THCA** (Fig. 9a), whereas none were found in the structures of the ligands **BCP** (Fig. 9b), Tamoxifen (Fig. 9c), or Erlotinib (Fig. 9d). For the ligands **THCA**, **BCP**, Tamoxifen, and Erlotinib, respectively, the molecular surface area stability parameter (MolSA) ranged from about 350 to 360 Å^2^ (Fig. 9a), 232–236 Å^2^ (Fig. 9b), 375–390 Å^2^ (Fig. 9c), and 400–405 Å^2^ (Fig. 9d). For the ligands **THCA**, **BCP**, Tamoxifen, and Erlotinib, respectively, the stability of the solvent accessible surface area (SASA) parameter ranged from 50 to 55 Å^2^ (Fig. 9a), 0–100 Å^2^ (Fig. 9b), 140–160 Å^2^ (Fig. 9c), and 160–200 Å^2^ (Fig. 9d). For the ligands **THCA**, **BCP**, Tamoxifen, and Erlotinib, the polar surface area (PSA) stability parameters were 1120–120 Å^2^ (Fig. 9a), 0 Å^2^ (Fig. 9b), 5–10 Å^2^ (Fig. 9c), and 64–72 Å^2^ (Fig. 9d)^,^ respectively.

#### 3.6.4 Thermodynamics properties

**Fig. 10:**
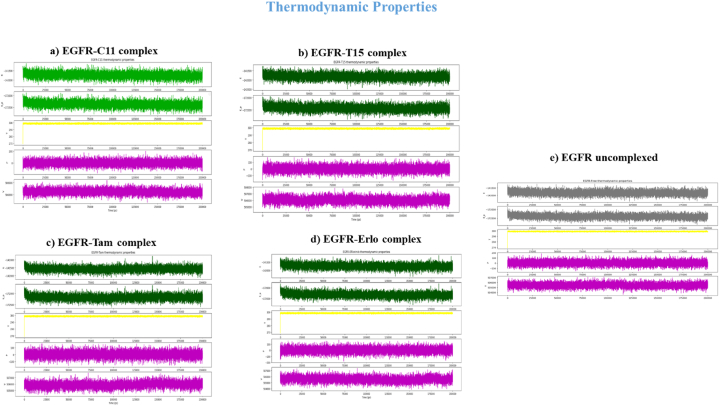
Thermodynamic properties timelines of Total Energy, Potential Energy, Temperature, Pressure and Volume for free EGFR (a) and the EGFR complexed w/C11 (b), T15 (c), Tamoxifen (d) and Erlotinib (e).

An overview of the quality of the MD simulation is given by the evaluation of thermodynamic parameters such as total energy (E), potential energy (EP), temperature (T), pressure (P), and volume (V) of protein-ligand systems. Table 4 and the graphical plots in Fig. 10(a-e) summarize the calculated mean values of thermodynamic properties and associated time scales that reflect the distribution of thermodynamic parameters E, EP, T, P, and V generated along a 200 ns trajectory from MD simulation runs for EGFR-C11 and EGFR-T15 samples, EGFR-Tamoxifen (Fig. 10c), EGFR-Erlotinib (Fig. 10d), and the free uncomplexed EGFR (Fig. 10e). Table 4 clearly demonstrates that the average values of E, EP, T, P, and V calculated for EGFR systems complexed with the ligands C11, T15, Tamoxifen, and Erlotinib were very close to the thermodynamic properties of the free EGFR protein. The stable distribution of the E, EP, T, P, and V parameter plots shown in Fig. 10a-e confirms these findings.

**Table 4:** Computed average values of Total Energy (E), Potential Energy (EP), Temperature (T), Pressure (P), and Volume (V) properties for the EGFR protein systems examined.

	EGFR uncomplexed	EGFR-C11	EGFR-T15	EGFR-Tam	EGFR-Erlo
Total energy (kcal/mol)	−141829.047	−141737.555	−141819.536	−141574.711	−141737.842
Potential energy (kcal/mol)	−172395.959	−172369.700	−172428.177	−172173.428	−172326.123
Temperature (K)	298.690	298.687	298.695	298.679	298.693
Pressure (bar)	1.356	1.269	1.562	0.974	1.031
Volume (Å^3^)	505443.948	506482.028	506029.727	505868.647	505560.214

As a whole, the collected data generated by the post-docking validation procedures, including MM-GBSA computations and MD simulations conducted in this study, can support the structural match of EGFR-TKD with the nine phytochemical samples isolated from the Cannabinoids and Terpenes. Thus, these substances could become future TKIs against breast and lung cancer for use alone or in combination with other drugs such as Tamoxifen and Erlotinib.

By applying virtual screening models and combining them with computer-aided drug design approaches and biophysical simulations, this study identified nine cannabis compounds (**CBDA**, **CBD**, **THCV**, **Δ-9-THC**, **Δ-8-THC**, **CBL**, **THCA**, **BCP**, **γ-Ele**) as promising candidates for novel epidermal growth factor receptor-tyrosine kinase inhibitors (EGFR-TKIs) in targeted cancer therapies. To further capitalize on these molecular scaffolds, further hit design and drug discovery efforts are warranted. To advance this concept and progress the lead compounds described in this work through the drug design pipeline, preliminary *in vitro* and *in vivo* assessments and analyses should be conducted. Finally, the advancements in scientific knowledge and technology have enabled the reintegration of natural products and their derivatives into drug design and development. This has provided an opportunity to overcome the technical barriers associated with detection, access, isolation, characterization, and valorization that had caused a decline in their use in the pharmaceutical industry beginning in the 1990s. Advances in analytical tools, engineering strategies applied to therapeutic research, and genome discovery in the agricultural sector have also facilitated this return. Consequently, the *Cannabis sativa* L. plant has been used as a model to challenge the stereotypes typically surrounding its recreational use in certain communities. Despite this, its production and consumption remain steady. In May 2021, the Moroccan state legalized the cultivation and export of *Cannabis sativa* L. for industrial, medical, and commercial purposes, excluding recreational purposes [95]. This could be a major shift in the region towards the legalization of the plant primarily for therapeutic use, considering Morocco's desirable climate, large territory, and convenient geographical location. Thus, this study aimed to identify and explore the potential opportunities in the field of *Cannabis sativa* L. to develop new medicinal agents, which could contribute to human safety and aid in the treatment of incurable diseases such as cancer.

## 4 Conclusion

Cancer is one of the leading causes of death worldwide, having accounted for more than 10 million deaths in 2020, equating to roughly one in every six deaths. Breast cancer, particularly in women, is the leading cause of cancer-related deaths, with an estimated 2.26 million lives lost to the disease in 2020. Lung cancer takes second place, responsible for 2.21 million deaths. In light of these alarming statistics, and the lack of effective treatment options, a study was conducted to explore the potential of phytochemicals derived from *Cannabis sativa* L to treat breast and lung cancers caused by abnormal enzymatic activity of EGFR-TKD (PDB ID:1M17). A total of fifty phytochemicals consisting of cannabinoids (C1–C12) and terpenes (T1-T38) were used to investigate their compatibility for binding interactions with the active pocket of EGFR-TKD (PDB ID:1M17). Tamoxifen and Erlotinib were used as references in the positive control *in silico* to identify the most promising lead candidate drugs for inhibiting the growth of breast and lung cancer cells.

Using Computer Aided Drug Design (CADD) methods and biophysical simulations, a multi-phase analysis of phytochemical compounds was conducted. Semi-flexible molecular docking simulations were then used to evaluate the ligands' affinity with the EGFR active site and rank them based on their most stable affinity energies in comparison to the reference drugs Tamoxifen and Erlotinib. Following this, an *in silico* predictive computational PK/PD models was developed combining Drug-like and ADME-Tox predictions with Osiris computations to select phytochemicals that met the criteria for oral bioavailability, pharmacokinetics, and pharmacodynamics. After screening the ligands, flexible molecular docking simulations were combined with MM-GBSA computations and molecular dynamics analysis to generate biophysical aspects related to Protein-ligand profiles interactions. This evaluation included parameters such as binding energies (BE), inhibition constants (Ki), free binding energies (ΔG_bind_), RMSD, RMSF, protein-ligand contacts, radius of gyration (rGyr), intramolecular hydrogen bonds (intraHB), molecular surface area (MolSA), solvent accessible surface area (SASA), polar surface area (PSA), total energy, potential energy, temperature. Results from the study indicated that the phytochemicals CBDA (Ki = 0.0175 μM, BE = −10.57 kcal/mol, ΔG_bind_ = −31.138 kcal/mol), CBD (Ki = 1.0281 μM, BE = −8.16 kcal/mol, ΔG_bind_ = −41.322 kcal/mol), THCV (Ki = 0.00967 μM, BE = −10.92 kcal/mol, ΔG_bind_ = −46.578 kcal/mol), Δ-9-THC (Ki = 0.0350 μM, BE = −10.16 kcal/mol, ΔG_bind_ = −40.446 kcal/mol), Δ-8-THC (Ki = 0.0665 μM, BE = −9.78 kcal/mol, ΔG_bind_ = −41.353 kcal/mol), CBL (Ki = 0.1182 μM, BE = −9.44, ΔG_bind_ = −35.748 kcal/mol), THCA (Ki = 0.0004115 μM, BE = −12.79 kcal/mol, ΔG_bind_ = −62.807 kcal/mol), BCP (Ki = 2.603 μM, BE = −7.61 kcal/mol, ΔG_bind_ = −33.980 kcal/mol), and γ-Ele (Ki = 3.650 μM, BE = −7.41 kcal/mol, ΔG_bind_ = −39.782 kcal/mol) displayed a more suitable conformational and pharmacological pattern to inhibit EGFR enzymatic activity when compared to the reference drugs Tamoxifen (Ki = 0.4000 μM, BE = −8.72 kcal/mol, ΔG_bind_ = −42.098 kcal/mol) and Erlotinib (Ki = 0.8253 μM, BE = −8.29 kcal/mol, ΔG_bind_ = −36.877 kcal/mol).

The analysis of drug-like properties related to bioavailability, toxicity risk, and ADME-Tox indicated that the chosen candidate drug compounds had very favorable drug-use characteristics. In contrast, the reference drugs Tamoxifen and Erlotinib displayed several aberrations and toxicity risks. This suggests that the proposed drugs' phytochemicals structures are likely to be more stable in the EGFR protein pocket, thus enabling them to reach their therapeutic target against breast and lung cancer cell growth with greater efficacy than Tamoxifen and Erlotinib. Molecular dynamics simulations conducted over a 200 ns trajectory further confirmed the stability of the target EGFR protein structure, as well as the ideal fit between the EGFR protein structure and the isolated samples of cannabinoids and terpenes phytochemicals . Consequently, the phytochemicals CBDA, CBD, THCV, Δ-9-THC, Δ-8-THC, CBL, THCA, BCP, and γ-Ele discussed in this study could be employed as structural keys for the development of new drugs that act as EGFR-TKIs for breast and lung cancer.

## Author contribution statement

Ossama Daoui: Conceived and designed the experiments; Performed the experiments; Analyzed and interpreted the data; Wrote the paper.

Suraj N. Mali, Kaouakeb Elkhattabi: Contributed reagents, materials, analysis tools or data.

Souad ElKhattabi, Samir Chtita: Conceived and designed the experiments; Contributed reagents, materials, analysis tools or data.

## Data availability statement

The raw/processed data required to reproduce these findings cannot be shared at this time as the data also forms part of an ongoing study.

## Consent for publication

All authors have agreed with the content of the manuscript.

## Funding

No funding was received for this project.

## Competing interests

The authors reported in the manuscript has no any conflict of interest.
