# Supplementary material for: Repositioning Cannabinoids and Terpenes as Novel EGFR-TKIs Candidates for Targeted Therapy Against Cancer: A virtual screening model using CADD and biophysical simulations
Source: Heliyon. 2023 Apr 17;9(4):e15545. doi: 10.1016/j.heliyon.2023.e15545 (PMC10148140; doi:10.1016/j.heliyon.2023.e15545)
Supplement: Multimedia component 1 [file mmc1.docx]

**Supplementary Information**

**Repositioning Cannabinoids and Terpenes as Novel EGFR-TKIs Candidates for Targeted Therapy Against Cancer: A Virtual Screening Model Using CADD and Biophysical Simulations**

**Ossama Daoui ^a, 1, *^, Suraj N. Mali ^b, 2^, Kaouakeb Elkhattabi ^c,3^, Souad Elkhattabi^a, 4^, and Samir Chtita ^d,5^**

^1^ [ossama.daoui@usmba.ac.ma](mailto:ossama.daoui@usmba.ac.ma), ^2^ [mali.suraj1695@gmail.com](mailto:mali.suraj1695@gmail.com), ^3^ [kaouakeb.elkhattabi@fmd.um5.ac.ma](mailto:kaouakeb.elkhattabi@fmd.um5.ac.ma), ^4^ [souad.elkhattabi@usmba.ac.ma](mailto:souad.elkhattabi@usmba.ac.ma), ^5^[samirchtita@gmail.com](mailto:samirchtita@gmail.com)

^a^ Laboratory of Engineering, Systems and Applications, National School of Applied Sciences, Sidi Mohamed Ben Abdellah-Fez University, P.O. Box 72, Fez, Morocco.

^b^ Department of Pharmaceutical Sciences and Technology, Birla Institute of Technology, Mesra, India-835215.

^c^ Department of Fundamental Sciences, Faculty of Dental Medicine, Mohammed V University in Rabat, Morocco

^d^ Laboratory of Analytical and Molecular Chemistry, Faculty of Sciences Ben M’Sik, Hassan II University of Casablanca, P.O. Box 7955, Casablanca, Morocco.

^*^Corresponding Author: [ossama.daoui@usmba.ac.ma](mailto:ossama.daoui@usmba.ac.ma)

**Molecular docking for virtual screening**

Molecular docking with Autodock Vina and Autodock 4 is important steps in virtual screening. Autodock Vina helps to rapidly identify potential binding sites on a target molecule by searching its binding sites, while Autodock 4 provides more accurate scoring of the binding energies of these sites. This combination of approaches helps to identify the most promising ligands for further study. Additionally, Autodock Vina and Autodock 4 can provide insight into the interactions between the ligand and the target molecule. This can help to determine the likely affinity and specific binding modes of the ligand, as well as potential adverse effects that may occur upon binding. The primary difference between AutoDock Vina and AutoDock 4 is that AutoDock Vina is a faster and more accurate algorithm than AutoDock 4. AutoDock Vina uses a global optimization algorithm to find the best structures, while AutoDock 4 uses a local optimization algorithm. Additionally, AutoDock Vina has an improved scoring function and a more robust search procedure than AutoDock 4. Lastly, AutoDock Vina allows for the docking of multiple ligands into the same receptor simultaneously, while AutoDock 4 does not.

*2.5. Conformational stability analysis of protein-ligand complexes*

- Predict binding energies and inhibitory activity concentrations

After selected the candidate drug compounds of isolated cannabinoids and cannabis terpenes that are able to inhibit EGFR-TKD enzymatic activity. Based on the density functional theory (DFT), these lead compounds are re-omptmized. This was done based on the Lee-Yang-Parr (B3LYP) function with the basis 6-31G (d,p) in the solvent water using the Gaussian 09 software [1]. All of this has been done to ensure proper equilibration of the system and to check the states of the bonding hydrogen atoms and protons in an aqueous environment similar to that of proteins.

Along with the help of AutoDock 4.2.6 software and Lamarckian genetic algorithm, the optimized drug molecules were re-docked inside the EGFR-TKD flexible active pocket [2]. We perform this phase to identify the best phytochemicals with the lowest binding energies (BE) and inhibition constants (Ki) against EGFR-TKD compared to the standard drugs Tamoxifen and Erlotinib. In this run, the prediction parameters were configured by applying the hybrid genetic algorithm with local search (GA-LS) and the number of energy evaluations was set to the maximum (25000000). The number of generations produced was set to the maximum (27000). The same grid box coordinates chosen in the molecular docking via AutoDock Vina 1.1.2 were implemented. The structure of EGFR-TKD protein (PDB ID:1M17) was prepared by adding polar hydrogens, calculating Kollman charge and Gasteiger charge. Identification of the chains of amino acid residues **Leu694**, **Val702**, **Ala719**, **Lys721**, **Met769**, **Asp776** and **Leu820** with Erlotinib interacts in the 1M17 complex as flexible reference points inside the active pocket of EGFR-TKD protein.

The remaining variables were all applied in the default setting. In each run, the top 5 clusters of docking pose for each single molecule against the protein receptor are listed after the structural alignment of the binding sites of the generated complexes. The clusters were ranked according to the highest score (lowest binding free energy) within each cluster. Consequently, from each cluster, the best flexible conformation of the molecule inside the flexible active pocket of the target protein is selected. The most stable conformation (lowest free binding energy and lowest distance RMSD cutoff) was identified as the best sample to analyze protein-ligand interactions in the present study.

- MM-GBSA Free Energies (ΔG_bind_) assessments

In a similar vein, using the MM-GBSA Prime tool provided in the Schrödinger 2020-3 package [3], we recalculated the novel binding modes in the protein-ligand systems (the three highest binding energy systems of each class of cannabinoids and terpenes). In this study, we applied this method to select the freest and selective ligands to interact and stabilize in the EGFR-TKD pocket by calculating the free binding energies (ΔG_bind_) for each system. ΔG_bind_ is produced by calculating a set of free binding parameters such as hydrogen bonding energy (ΔGbind H-bond), van der Waals energy (ΔG_bind_ vdW), covalent energy (ΔG_bind_ covalent), coulombic energy (ΔG_bind_ Coulomb), and lipophilic energy (ΔG_bind_ Lipo.), the generalized Born electrostatic solvation energy (ΔG_bind_ Solv_GB), and the packing energy (ΔG_bind_ Coulomb) [4, 5].

The sum of the Prime MM-GBSA computations derived from the optimization of the energies of the complexes (E) and ligands (E ligands) generates the binding free energy (ΔGbind) of the protein-ligand complexes, with ΔG_bind_=E_complex_ (minimized) + E_ligand_ (minimized) –E_free receptor_ (minimized) [4].

- Molecular dynamics simulations

Molecular dynamics and Brownian dynamics approaches are among the most powerful computer simulations of molecular systems composed of large groups of molecules at a time [6]. These types of approaches allow the trajectories of atoms and molecules to be tracked from the computational solution of Newton's equations of motion for biological systems composed of interacting particles over a time frame. In the present study, we have focused our attention on evaluating the structural and dynamical stability of the free and complexed EGFR-TKD protein (PDB:1M17) with cannabinoid and terpenes samples as future candidates for cancer drugs. Test samples were taken on the basis of the free binding energy values of the lead molecules to EGFR-TKD obtained by MM-GBSA free binding energy (ΔG_bind_) computations. Test samples were taken based on the free binding energy values of the lead phytochemicals to EGFR-TKD provided from MM-GBSA free binding energy computations (ΔG_bind_). All MDS operations in this work were performed using the Desmond package available in the Schrödinger 2020-3 academic software program according to the same strategy used in our previous work [1, 4, 7]. The MDS run time was set to 200 ns for each individual discrete system, the systems sampled and optimized using the OPLS3e force field and solvated in the SPC model. The orthorhombic simulation box was prepared by setting the minimum distance between the protein boundary surface and the protein edge surface at 10 Å. Sodium and chloride ions were added to neutralize the charge of the systems and adjust the physiological salt concentration to 0.15 M. The energies of the systems were optimized to 2000 steps using a Columbian interaction with 9 Å cutoffs and a 0.8 Å lattice phase. The smooth particle mesh Ewald method with a tolerance of 1E-09 was used to resolve the long-range electrostatic interactions. Using the Nose-Hoover algorithm and the Martina-Tobias-Klein method, the systems were slowly heated to a standard temperature of 310 K and a pressure of 1.013 bar under an isothermal-isobaric ensemble (NPT). The MDS trajectory run was set to 20000 frames per simulated trajectory, recording intervals of 1.2 ps for energy, and 10 ps for trajectory, 1 ps and 2 fs for the systems relaxation.

**Table S1.** Data of phytochemicals and terpenes of Cannabis sativa L.

| **Cat.** | **PubChem CID** | **ID Name** | **Name** | **IUPAC Name/2D Structure** |
| --- | --- | --- | --- | --- |
| **Isolated cannabinoids** | 160570 | **C1**: CBDA | Cannabidiolic acid | 2,4-dihydroxy-3-[(1R,6R)-3-methyl-6-prop-1-en-2-ylcyclohex-2-en-1-yl]-6-pentylbenzoic acid |
|  |  |  |  |  |
|  | 6449999 | **C2 :** CBGA | Cannabigerolic Acid | 3-[(2E)-3,7-dimethylocta-2,6-dienyl]-2,4-dihydroxy-6-pentylbenzoic acid |
|  |  |  |  |  |
|  | 5315659 | **C3**: CBG | Cannabigerol | 2-[(2E)-3,7-dimethylocta-2,6-dienyl]-5-pentylbenzene-1,3-diol |
|  |  |  |  |  |
|  | 644019 | **C4**: CBD | Cannabidiol | 2-[(1R,6R)-3-methyl-6-prop-1-en-2-ylcyclohex-2-en-1-yl]-5-pentylbenzene-1,3-diol |
|  |  |  |  |  |
|  | 93147 | **C5** : THCV | Tetrahydrocannabivarin | (6aR,10aR)-6,6,9-trimethyl-3-propyl-6a,7,8,10a-tetrahydrobenzo[c]chromen-1-ol |
|  |  |  |  |  |
|  | 2543 | **C6**: CNB | Cannabinol | 6,6,9-trimethyl-3-pentylbenzo[c]chromen-1-ol |
|  |  |  |  |  |
|  | 16078 | **C7**: Δ-9-THC | Dronabinol | (6aR,10aR)-6,6,9-trimethyl-3-pentyl-6a,7,8,10a-tetrahydrobenzo[c]chromen-1-ol |
|  |  |  |  |  |
|  | 2977 | **C8**: Δ-8-THC | Delta-8-Tetrahydrocannabinol | 6,6,9-trimethyl-3-pentyl-6a,7,10,10a-tetrahydrobenzo[c]chromen-1-ol |
|  |  |  |  |  |
|  | 30607 | **C9**: CBL | Cannabicyclol | 9,13,13-trimethyl-5-pentyl-8-oxatetracyclo[7.4.1.02,7.012,14]tetradeca-2,4,6-trien-3-ol |
|  |  |  |  |  |
|  | 30219 | **C10**: CBC | Cannabichromene | 2-methyl-2-(4-methylpent-3-enyl)-7-pentylchromen-5-ol |
|  |  |  |  |  |
|  | 98523 | **C11**: THCA | Delta9-tetrahydrocannabinolic acid | (6aR,10aR)-1-hydroxy-6,6,9-trimethyl-3-pentyl-6a,7,8,10a-tetrahydrobenzo[c]chromene-2-carboxylic acid |
|  |  |  |  |  |
|  | 3084339 | **C12**: CBCA | Cannabichromenic Acid | 5-hydroxy-2-methyl-2-(4-methylpent-3-enyl)-7-pentylchromene-6-carboxylic acid |
|  |  |  |  |  |
| **Cannabis Terpenes** | 6654 | **T1**: α-pinene | Alpha-Pinene | 2,6,6-trimethylbicyclo[3.1.1]hept-2-ene |
|  |  |  |  |  |
|  | 6616 | **T2**: Camphene | Camphene | 2,2-dimethyl-3-methylidenebicyclo[2.2.1]heptane |
|  |  |  |  |  |
|  | 14896 | **T3**: β-pinene | Beta-Pinene | 6,6-dimethyl-2-methylidenebicyclo[3.1.1]heptane |
|  |  |  |  |  |
|  | 31253 | **T4**: β-myrcene | Myrcene | 7-methyl-3-methylideneocta-1,6-diene |
|  |  |  |  |  |
|  | 26049 | **T5**: δ-3-carene | Delta-3-Carene | 3,7,7-trimethylbicyclo[4.1.0]hept-3-ene |
|  |  |  |  |  |
|  | 7462 | **T6**: α-terpinene | Alpha-Terpinene | 1-methyl-4-propan-2-ylcyclohexa-1,3-diene |
|  |  |  |  |  |
|  | 7463 | **T7**: p-cymene | para-Cymene | 1-methyl-4-propan-2-ylbenzene |
|  |  |  |  |  |
|  | 440917 | **T8**: d-limonene | D-Limonene | (4R)-1-methyl-4-prop-1-en-2-ylcyclohexene |
|  |  |  |  |  |
|  | 5320250 | **T9**: ocimene | cis-beta-Ocimene | 3,7-dimethylocta-1,3,6-triene |
|  |  |  |  |  |
|  | 7461 | **T10**: γ-terpinene | Gamma-Terpinene | 1-methyl-4-propan-2-ylcyclohexa-1,4-diene |
|  |  |  |  |  |
|  | 11463 | **T11**: Terpinolene | Isoterpinene | 1-methyl-4-propan-2-ylidenecyclohexene |
|  |  |  |  |  |
|  | 6549 | **T12**: linalool | Linalool | 3,7-dimethylocta-1,6-dien-3-ol |
|  |  |  |  |  |
|  | 170833 | **T13**: (−)-isopulegol | Isopulegol | (1R,2S,5R)-5-methyl-2-prop-1-en-2-ylcyclohexan-1-ol |
|  |  |  |  |  |
|  | 637566 | **T14**: geraniol | Geraniol | (2E)-3,7-dimethylocta-2,6-dien-1-ol |
|  |  |  |  |  |
|  | 5281515 | **T15**: β-caryophyllene | Beta-Caryophyllene | (1R,4E,9S)-4,11,11-trimethyl-8-methylidenebicyclo[7.2.0]undec-4-ene |
|  |  |  |  |  |
|  | 5281520 | **T16**: α-humulene | Alpha-Humulene | (1E,4E,8E)-2,6,6,9-tetramethylcycloundeca-1,4,8-triene |
|  |  |  |  |  |
|  | 5284507 | **T17**: nerolidol | Nerolidol | (6E)-3,7,11-trimethyldodeca-1,6,10-trien-3-ol |
|  |  |  |  |  |
|  | 227829 | **T18**: (−)-guaiol | Guaiol | 2-[(3S,5R,8S)-3,8-dimethyl-1,2,3,4,5,6,7,8-octahydroazulen-5-yl]propan-2-ol |
|  |  |  |  |  |
|  | 348291297 | **T19**: (−)-α-bisabolol | (-)-Alpha-Bisabolol | (2S)-6-methyl-2-[(1S)-4-methylcyclohex-3-en-1-yl]hept-5-en-2-ol |
|  |  |  |  |  |
|  | 2758 | **T20**: cineol | Cineole | 1,3,3-trimethyl-2-oxabicyclo[2.2.2]octane |
|  |  |  |  |  |
|  | 6997371 | **T21**: fenchol | Fenchyl Alcohol | (1R,2R,4S)-1,3,3-trimethylbicyclo[2.2.1]heptan-2-ol |
|  |  |  |  |  |
|  | 6549 | **T22**: linalol | Linalool | 3,7-dimethylocta-1,6-dien-3-ol |
|  |  |  |  |  |
|  | 64685 | **T23**: borneol | Borneol | 1,7,7-trimethylbicyclo[2.2.1]heptan-2-ol |
|  |  |  |  |  |
|  | 17100 | **T24**: α-terpineol | alpha-Terpineol | 2-(4-methylcyclohex-3-en-1-yl)propan-2-ol |
|  |  |  |  |  |
|  | 6432312 | **T25**: γ-elemene | Gamma-Elemene | (1S,2S)-1-ethenyl-1-methyl-4-propan-2-ylidene-2-prop-1-en-2-ylcyclohexane |
|  |  |  |  |  |
|  | 86608 | **T26**: α-bergomotene | Alpha-Bergamotene | 2,6-dimethyl-6-(4-methylpent-3-enyl)bicyclo[3.1.1]hept-2-ene |
|  |  |  |  |  |
|  | 5281517 | **T27**: β-Farnesene | Beta-Farnesene | (6E)-7,11-dimethyl-3-methylidenedodeca-1,6,10-triene |
|  |  |  |  |  |
|  | 442393 | **T28**: β-eudesmene | Beta-Eudesmene | (3R,4aS,8aR)-8a-methyl-5-methylidene-3-prop-1-en-2-yl-1,2,3,4,4a,6,7,8-octahydronaphthalene |
|  |  |  |  |  |
|  | 9855795 | **T29**: valencene | Valencene | (3R,4aS,5R)-4a,5-dimethyl-3-prop-1-en-2-yl-2,3,4,5,6,7-hexahydro-1H-naphthalene |
|  |  |  |  |  |
|  | 6432384 | **T30**: α-bulnesene | Alpha.-Bulnesene | (3R,5S)-3,8-dimethyl-5-prop-1-en-2-yl-1,2,3,3a,4,5,6,7-octahydroazulene |
|  |  |  |  |  |
|  | 5281516 | **T31**: farnesene | Alpha-Farnesene | (3E,6E)-3,7,11-trimethyldodeca-1,3,6,10-tetraene |
|  |  |  |  |  |
|  | 6450812 | **T32**: β-gurjunene | Beta-Gurjunene | (1aR,4R,4aR,7aR,7bR)-1,1,4-trimethyl-7-methylidene-2,3,4,4a,5,6,7a,7b-octahydro-1aH-cyclopropa[e]azulene |
|  |  |  |  |  |
|  | 522296 | **T33**: eudesma-3,7(11)-diene | Eudesma-3,7(11)-diene | 5,8a-dimethyl-3-propan-2-ylidene-1,2,4,4a,7,8-hexahydronaphthalene |
|  |  |  |  |  |
|  | 519743 | **T34**: seychellene | Seychellene | 3,6,8-trimethyl-2-methylidenetricyclo[5.3.1.03,8]undecane |
|  |  |  |  |  |
|  | 520383 | **T35**: δ-selinene | Delta.-Selinene | 4,8a-dimethyl-6-propan-2-yl-2,3,7,8-tetrahydro-1H-naphthalene |
|  |  |  |  |  |
|  | 6432005 | **T36**: γ-eudesmol | Gamma-Eudesmol | 2-[(2R,4aR)-4a,8-dimethyl-2,3,4,5,6,7-hexahydro-1H-naphthalen-2-yl]propan-2-ol |
|  |  |  |  |  |
|  | 92762 | **T37**: α-eudesmol | Alpha-Eudesmol | 2-[(2R,4aR,8aR)-4a,8-dimethyl-2,3,4,5,6,8a-hexahydro-1H-naphthalen-2-yl]propan-2-ol |
|  |  |  |  |  |
|  | 90785 | **T38**: bulnesol | Bulnesol | 2-(3,8-dimethyl-1,2,3,3a,4,5,6,7-octahydroazulen-5-yl)propan-2-ol |
|  |  |  |  |  |

**Table S2.** Analysis of binding affinities of ligands (C1-C12 and T1-T38, as well as the reference drugs Tamoxifen and Erlotinib) inside the active pocket of EGFR-TKD.

| **Category of phytoconstituents** | **Ligand name** | **Binding affinities**  (Kcal/mol) |
| --- | --- | --- |
| Isolated cannabinoids | C1 | -9.9 |
|  | C2 | 8.1 |
|  | C3 | -7.2 |
|  | C4 | -9.8 |
|  | C5 | -9.9 |
|  | C6 | -10.8 |
|  | C7 | -10.7 |
|  | C8 | -10.1 |
|  | C9 | -10.0 |
|  | C10 | -8.4 |
|  | C11 | -11.3 |
|  | C12 | -8.5 |
| Cannabis Terpenes | T1 | -5.6 |
|  | T2 | -5.5 |
|  | T3 | -5.6 |
|  | T4 | -5.7 |
|  | T5 | -5.7 |
|  | T6 | -5.7 |
|  | T7 | -5.5 |
|  | T8 | -5.7 |
|  | T9 | -5.6 |
|  | T10 | -5.6 |
|  | T11 | -5.5 |
|  | T12 | -6.0 |
|  | T13 | -6.0 |
|  | T14 | -6.1 |
|  | T15 | -8.4 |
|  | T16 | -6.7 |
|  | T17 | -8.8 |
|  | T18 | -7.2 |
|  | T19 | -7.2 |
|  | T20 | -5.6 |
|  | T21 | -6.1 |
|  | T22 | -6.0 |
|  | T23 | -5.6 |
|  | T24 | -5.9 |
|  | T25 | -8.4 |
|  | T26 | -7.1 |
|  | T27 | -7.3 |
|  | T28 | -8.5 |
|  | T29 | -7.2 |
|  | T30 | -7.4 |
|  | T31 | -7.3 |
|  | T32 | -6.8 |
|  | T33 | -7.6 |
|  | T34 | -6.2 |
|  | T35 | -7.3 |
|  | T36 | -7.3 |
|  | T37 | -7.9 |
|  | T38 | -7.9 |
| Standard drug | Tamoxifen | -9.4 |
|  | Erlotinib | -8.2 |

**Table S3**. ADME-Tox pharmacokinetic parameters of candidate drug compounds

|  | **models** | | | | | | | | | | | | | | | | | | | | |  |
| --- | --- | --- | --- | --- | --- | --- | --- | --- | --- | --- | --- | --- | --- | --- | --- | --- | --- | --- | --- | --- | --- | --- |
|  | **Absorption** | | **Distribution** | | | | **Metabolism** | | | | | | | **Excretion** | **Toxicity** | | | | | | |  |
| **Properties** | Intestinal absorption  (human) | Skin Permeability | VDss (human) | Fraction unbound (human) | BBB permeability | CNS permeability | Cytochrome P450 (CYP450) | | | | | | | Total clearance | AMES toxicity | | ORAT (LD_50_) | | Hepatotoxicity | Max. tolerated dose (human) | |  |
|  |  |  |  |  |  |  | Substrate | | Inhibitor | | | | |  |  |  |  |  |  |  |  |  |
|  |  |  |  |  |  |  | 2D6 | 3A4 | 1A2 | 2C19 | 2C9 | 2D6 | 3A4 |  | |  | |  |  | |  | |
| **Unity** | % | log Kp | Log L kg-1 | Numeric | Log BB | Log PS | yes/no | | | | | | | log  mL min^-1^ kg^-1^ | yes/no | | mol/kg | | yes/no | log mg/kg/day | |  |
| C1 | 98.61 | -2.73 | -1.412 | 0.09 | -0.93 | -1.97 | No | No | No | No | No | No | No | 0.98 | | No | | 2.47 | No | | 0.52 | |
| C4 | 91.02 | -2.88 | 0.76 | 0.06 | 0.05 | -1.40 | No | Yes | Yes | No | No | No | No | 1.13 | | No | | 2.29 | No | | -0.37 | |
| C5 | 92.56 | -2.75 | 0.78 | 0.06 | 0.30 | -1.80 | No | No | Yes | Yes | No | No | No | 0.93 | | No | | 2.18 | No | | -0.152 | |
| C6 | 93.18 | -2.75 | 0.65 | 0.01 | 0.60 | -1.06 | No | Yes | Yes | Yes | Yes | No | No | 0.95 | | Yes | | 2.35 | No | | 0.26 | |
| C7 | 91.87 | -2.75 | 0.85 | 0 | 0.42 | -1.69 | No | Yes | Yes | Yes | No | No | Yes | 0.98 | | No | | 2.23 | No | | -0.15 | |
| C8 | 91.87 | -2.78 | 0.85 | 0 | 0.42 | -1.69 | No | Yes | Yes | Yes | No | No | Yes | 0.97 | | No | | 2.23 | No | | -0.15 | |
| C9 | 91.92 | -2.80 | 0.86 | 0 | 0.47 | -1.61 | No | Yes | Yes | Yes | No | No | No | 0.55 | | No | | 2.33 | No | | -0.52 | |
| C11 | 97.56 | -2.73 | -1.22 | 0.09 | -0.13 | -1.89 | No | No | No | No | No | No | No | 0.74 | | No | | 2.77 | No | | 0.56 | |
| T15 | 95.30 | -1.59 | 0.65 | 0.26 | 0.74 | -2.18 | No | No | No | No | No | No | No | 1.08 | | No | | .1.67 | No | | 0.48 | |
| T25 | 95.43 | -1.27 | 0.59 | 0.14 | 0.79 | -1.68 | No | No | No | No | No | No | No | 1.41 | | No | | 1.58 | No | | 0.19 | |
| T28 | 94.76 | -1.89 | 0.60 | 0.09 | 0.81 | -1.40 | No | Yes | Yes | No | No | No | No | 1.17 | | Yes | | 1.61 | No | | -0.42 | |
| Tam | 97.8 | -2.73 | 0.40 | 0.16 | 1.40 | -1.61 | No | Yes | Yes | No | No | Yes | No | 0.60 | | Yes | | 2.17 | No | | 0.52 | |
| Erl | 95.26 | -2.73 | -0.27 | 0.14 | -1.01 | -3.46 | No | Yes | Yes | Yes | Yes | No | Yes | 0.64 | | No | | 2.81 | Yes | | 0.91 | |
| VDss: Volume of Distribution by the Steady-State method, BBB: Blood-Brain Barrier, CNS: Central Nervous System. ORAT: Oral Rat Acute Toxicity.  Molecules marked in red have potential toxicity. | | | | | | | | | | | | | | | | | | | | | |  |

**Table S4.** Interactions of lead inhibitors with the EGFR-TKD.

|  | **EGFR-TKIs non-covalent Interactions** | | | | | | | | |  | **BE** | **Ki** | **ΔG_bind_** |
| --- | --- | --- | --- | --- | --- | --- | --- | --- | --- | --- | --- | --- | --- |
|  | **Hydrogen Bond** | | | **Hydrophobic** | | | | **Electrostatic** | | **Other** | **kcal/mol** | **(µM)**  **±0.001** | **Prime MM-GBSA**  **(kcal/mol)** |
| **Flexible ligand** | **Conventional** | | **Carbon** | **π-Sigma** | **Alkyl** | **π-Alkyl** | **π-π T-shaped** | **π-Cation** | **π-Anion** | **π-Sulfur** |  |  |  |
| **C1** | 3 : THR830 (2.0 Å) and **LYS721** (2.12, 2.20 Å), | | ND | 2: **VAL702** (3.89, 3.33 Å) | 4: **LEU694** (3.85 Å), **LYS721** (4.49 Å), **LEU820** (5.03 Å) and **VAL702** (4.80 Å) | 4:PHE699 (3.87, 4.47, 4.87Å) and **LEU820** (5.01 Å) | ND | ND | ND | ND | -10.57 | 0.0175 | -31.138 |
| **C4** | 1: **LEU694** (2.15 Å) | | ND | 1: **VAL702** (3.88 Å) | 5: **LYS721** (4.36 Å), CYS773 (3.27 Å), LEU768 (4.55 Å), PRO770 (4.84 Å) and **LEU694** (3.76 Å) | 2: PHE699 (3.83, 4.25, 5.24 Å) and LEU82 (5.27 Å) | ND | ND | ND | ND | -8.16 | 1.0281 | -41.322 |
| **C5** | 2: THR766 (2.24 Å) and **LYS721** (2.19 Å) | | ND | ND | 16: **ALA719** (5.30, 5.15, 4.37Å), **MET769** (5.34, 4.97 Å), **LEU820** (4.26, 4.34, 4.80 Å), **VAL702** (4.16, 4.03, 4.79 Å), LEU768 (4.91 Å), **LEU694** (4.51 Å), LEU764 (4.53 Å), **LYS721** (5.04, 5.44 Å), | 3:PHE699 (4.73 Å), **ALA719** (4.71 Å), **VAL702** (4.95 Å), **LYS721** (4.55 Å) | ND | ND | ND | 2; **MET769** (2.99, 5.71 Å) | -10.92 | 0.00967 | -46.578 |
| **C7** | 2: **LYS721** (2.20, 2.17 Å) | | ND | 3: PHE699 (3.22, 3.99 Å), **VAL702** (3.97 Å) | 4: MET742 (4.96 Å), **VAL702** (3.80, 4.58 Å), **LYS721** (5.36 Å) | 2: **ALA719** (4.42 Å), **LYS721** (4.90 Å) | ND | ND | ND | **MET769** (4.70 Å) | -10.16 | 0.0350 | -40.446 |
| **C8** | 1: **LYS721** (2.20 Å) | | ND | 4: PHE699 (3.79, 3.68 Å), **VAL702** (3.97 Å), **LEU820** (3.98 Å) | 5 : VAL 702 (4.43, 4.95 Å), CYS773 (4.36 Å ), **LEU694** (3.94 Å ), **LEU820** (5.09 Å), | 2 : PHE699 (4.57 Å), **ALA719** (5.34 Å) | ND | ND | ND | ND | -9.78 | 0.0665 | -41.353 |
| **C9** | 2: ASP831 (2.20 Å), **LYS721** (2.21 Å) | | ND | 2 : PHE699 (3.92 Å), **VAL702** (3.59 Å)° | 6 : CYC773 (3.98, 4.50 Å), **LEU820** (4.02 Å), MET742 (4.74 Å), LEU764 (5.20 Å), **VAL702** (4.67 Å) | 3 : PHE699 (5.22 Å), **LYS721** (4.88 Å), LEU82 (5.36 Å) | ND | ND | ND | ND | -9.44 | 0.1182 | -35.748 |
| **C11** | 7 : THR830 (1.86, 1.85 Å), ASP831 (2.16, 2.99 Å), GLU738 (1.73 Å), **LYS721** (2.21, 1.66 Å) | |  |  | 16 : **ALA719** (4.99, 3.18 Å), **MET769** (5.44, 4.60 Å), **LEU820** (4.17, 4.60 Å), CYS773 (4.52 Å), **VAL702** (4.94, 4.10, 5.15 Å), VAL768 (4.60 Å), **LEU694** (5.46 Å), LEU723 (5.06 Å), ILE735 (4.88 Å), **LYS721** (5.32 Å), **LEU820** (4.70 Å) | 2 : **VAL702** (4.37 Å), **LYS721** (4.40 Å) |  |  | 1 : ASP831 (4.70 Å) |  | **-12.79** | **0.4115 E^-3^** | -62.807 |
| **T15** | ND | | ND | ND | 16 : **ALA719** (3.72, 3.99, 4.81 Å), **VAL702** (4.77, 4.60, 3.75, 4.18 Å), **LYS721** (5.32, 4.74, 4.07), **MET769** (4.92, 4.45 Å), **LEU820** (4.96, 4.92 Å). | ND | ND | ND | ND | ND | -7.61 | 2.603 | -33.980 |
| **T25** | ND | | ND | ND | 14 : **ALA719** (4.60, 318, 3.72 Å), **VAL702** (3.27, 5.20, 4.23 Å), **LYS721** (3.50, 4.46 Å), **MET769** (5.17, 4.85 Å), **LEU820** (4.06, 4.45 Å), LEU768 (4.64 Å), **LEU694** (5.20 Å). | ND | ND | ND | ND | ND | -7.41 | **3.650** | -39.782 |
| **Tamoxifen** | ND | | 1 : ASN818 (2.93 Å) | 1 : ALA 719 (3.73 Å) | 5 : **ALA719** (4.11 Å), **LEU694** (4.83 Å), **VAL702** (5.22 Å), **MET769** (4.30 Å), **LEU820** (4.84 Å) | 6 : **VAL702** (4.98, 4.58, 5.23 Å), **LYS721** (4.98, 4.02 Å), **LEU820** (4.89 Å) | 1 : PHE699 (5.13 Å) | 2 : **LYS721** (4.26, 4.96 Å) |  | ND | -8.72 | 0.4000 | -42.098 |
| **Erlotinib** | 4 **: MET769 (2.65 Å),** **LYS721 (1.78, 2.22, 2.06 Å)**. | | **2 : ASP831 (3.51** Å), GLU738 (2.83 Å) | **1 : VAL702 (3.91 Å)** | **-** | 9 : **LEU694 (5.38, 4.17, 5.45 Å)**, **ALA719 (5.24, 4.88 Å)**, LEU768 (5.41 Å), LEU769 (5.06 Å), **MET769** Å (4.82 Å), **LEU820 (5.14 Å)** | ND | ND | ND | ND | -8.29 | 0.8253 | -36.877 |
| **Reference flexible amino acid residues** | | **Leu694**, **Val702**, **Ala719**, **Lys721**, **Met769**, **Asp776** and **Leu820** | | | | | | | | | | | |
| ND: Not detected. | | | | | | | | | | | | | |

| 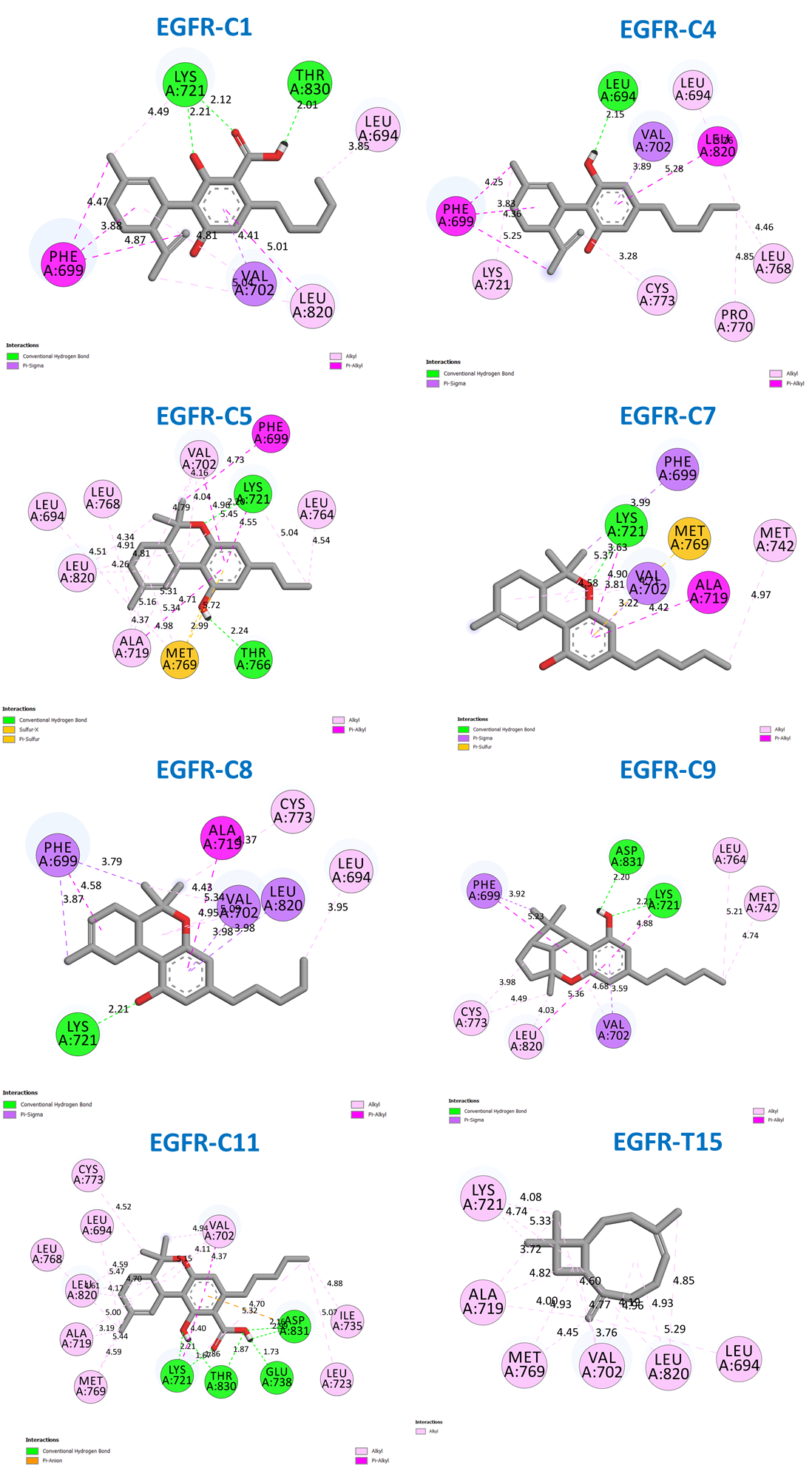 |
| --- |
| 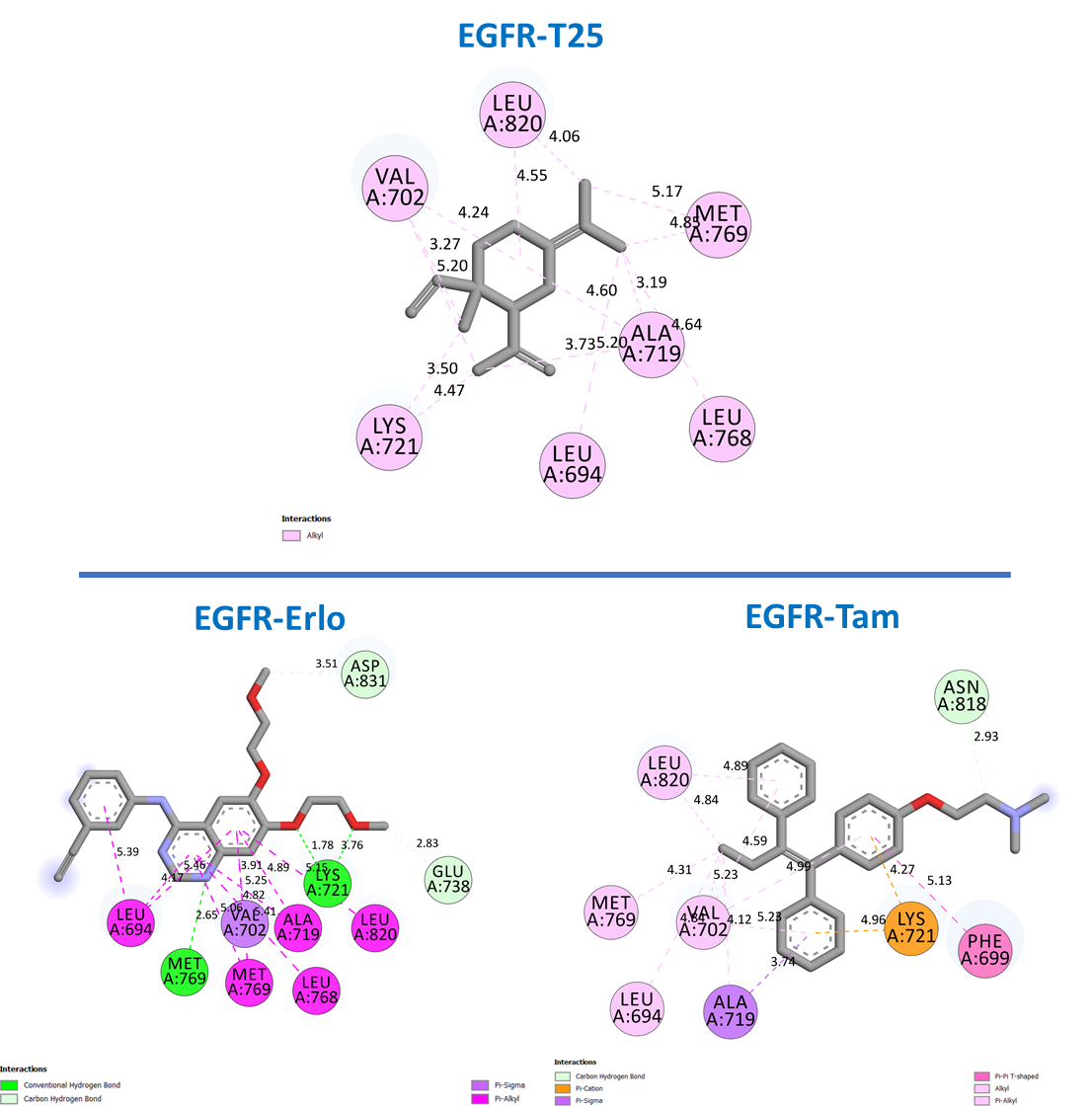 |

**Figure S1**. Molecular docking visualization of the interactions of TKIs with the EGFR receptor.

| 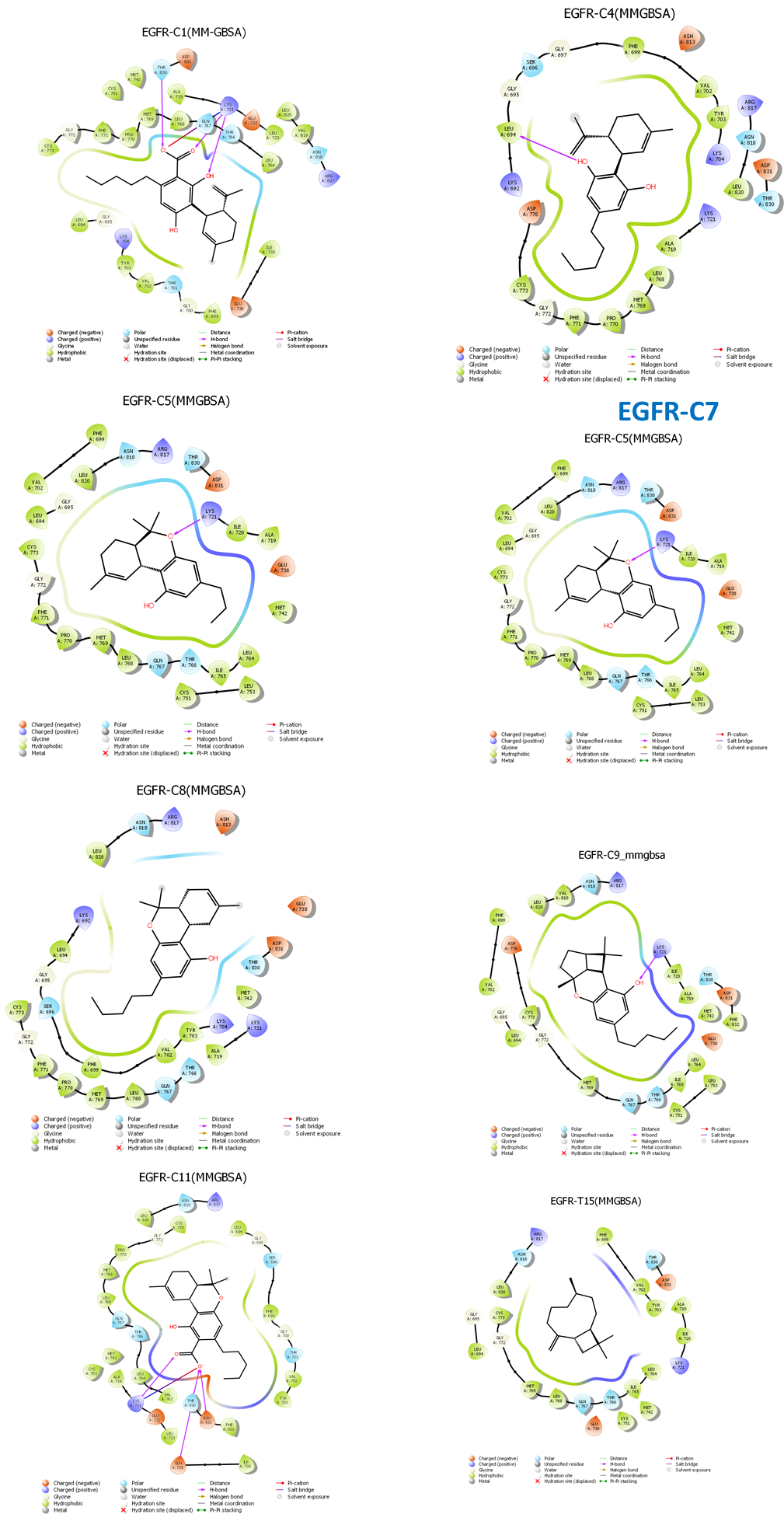 |
| --- |
| 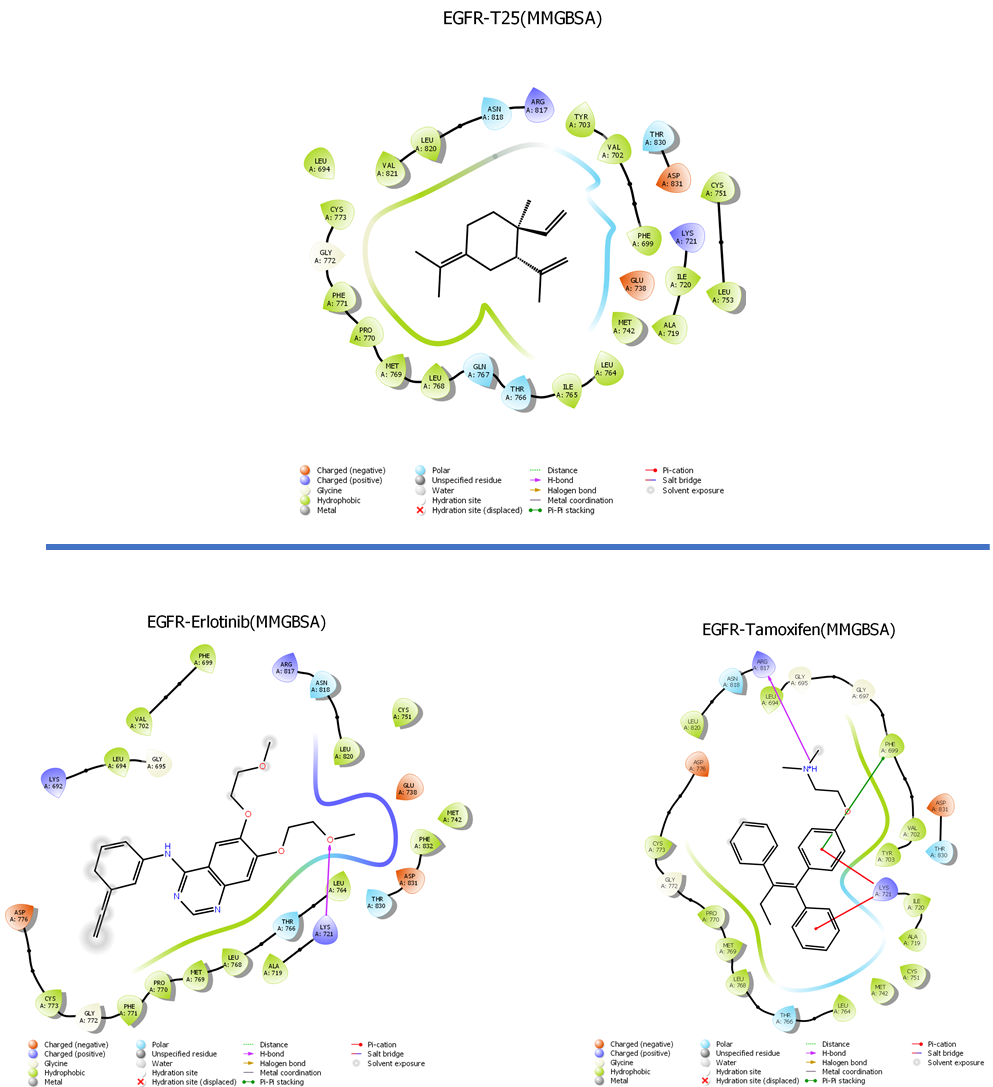 |

**Figure S2**. Prime MM-GBSA visualization of the interactions of TKIs with the EGFR receptor.

**References**

1. Daoui O, Elkhattabi S, Chtita S (2022) Rational design of novel pyridine-based drugs candidates for lymphoma therapy. J Mol Struct 1270:133964

2. Castro-Alvarez A, Costa AM, Vilarrasa J (2017) The performance of several docking programs at reproducing protein–macrolide-like crystal structures. Molecules 22:136

3. Li J, Abel R, Zhu K, et al (2011) The VSGB 2.0 model: a next generation energy model for high resolution protein structure modeling. Proteins Struct Funct Bioinforma 79:2794–2812

4. Daoui O, Elkhattabi S, Chtita S (2022) Rational identification of small molecules derived from 9, 10-dihydrophenanthrene as potential inhibitors of 3CLpro enzyme for COVID-19 therapy: a computer-aided drug design approach. Struct Chem 33:1667–1690

5. Daoui O, Elkhattabi S, Chtita S (2022) Rational design of novel pyridine-based drugs candidates for lymphoma therapy. J Mol Struct 1270:133964. https://doi.org/10.1016/j.molstruc.2022.133964

6. Wang Y, Ribeiro JML, Tiwary P (2020) Machine learning approaches for analyzing and enhancing molecular dynamics simulations. Curr Opin Struct Biol 61:139–145

7. Ouassaf M, Daoui O, Alam S, et al (2022) Pharmacophore-based virtual screening, molecular docking, and molecular dynamics studies for the discovery of novel FLT3 inhibitors. J Biomol Struct Dyn 0:1–13. https://doi.org/10.1080/07391102.2022.2123403
